# Mapping the secondary response to traumatic brain injury using spatial transcriptomics shows acute 4-aminopyridine treatment mitigates axonal and molecular pathology

**DOI:** 10.1186/s40478-025-02219-1

**Published:** 2026-01-08

**Authors:** Genevieve M. Sullivan, Kryslaine L. Radomski, Shaoqiu He, Matthew D. Wilkerson, Clifton L. Dalgard, Camille Alba, Xiaomei Zi, Martin L. Doughty, Regina C. Armstrong

**Affiliations:** 1 Department of Anatomy, Physiology and Genetics in the School of Medicine at the Uniformed Services University of the Health Sciences, Bethesda, 4301 Jones Bridge Rd., Bethesda, MD 20814 USA; 2 Military Traumatic Brain Injury Initiative (MTBI2), Bethesda, MD USA; 3 Center for Military Precision Health, Bethesda, MD USA; 4 The Henry M. Jackson Foundation for the Advancement of Military Medicine, Inc. (HJF), Bethesda, MD USA

**Keywords:** Genome-wide spatial transcriptomics, 4-aminopyridine, Potassium channel, Traumatic brain injury, Axon damage, Myelin

## Abstract

Damage to long myelinated axons of white matter tracts is a hallmark pathology resulting from traumatic brain injury (TBI) forces and secondary injury processes. 4-aminopyridine (4-AP) is an FDA-approved Kv1 potassium channel inhibitor designed to mitigate axon dysfunction. We examined repurposing 4-AP as an acute TBI treatment using clinically-oriented neuropathology of axon damage combined with unbiased genome-wide spatial transcriptomics for comprehensive analysis of secondary injury processes. Adult male and female mice received a non-penetrating impact TBI with 4-AP (i.p., *b.i.d*) on days 1–7 post-injury. Along corpus callosum (CC) axons, TBI disrupted node of Ranvier domains, exposing the putative 4-AP target of mislocalized Kv1 channels (*p* < 0.005). Clinically reasonable 4-AP dosing (0.5 mg/kg) reduced nodal Nav1.6 channel loss (*p* < 0.05) and Caspr heminode formation (*p* < 0.005) after injury. Quantification of β-amyloid precursor protein immunolabeling showed significantly reduced CC axon damage at 4-AP doses of 0.5 mg/kg and 5 mg/kg (each *p* < 0.005). 4-AP safety, based on potential seizure risk after TBI, was unaltered with vehicle or 0.5 mg/kg 4-AP, while the 5 mg/kg dose induced seizure behavior in sham and TBI groups (*p* < 0.0001). Spatial transcriptomics mapped molecular signatures to tissue pathology. TBI increased axonal injury response genes in the CC and in motor and somatosensory cortex sites of CC projection neurons. TBI induced disease-associated glial phenotypes that mapped predominantly within the CC. TBI increased pathway expression for immune and vascular functions, neuron and glial cell signaling, and cellular dyshomeostasis, while reducing expression in myelination-related pathways. Gene expression analysis of 4-AP treatment (0.5 mg/kg) indicated potassium channel target engagement and increased neuroaxonal activity, along with dampened secondary injury responses. Collectively, these findings reveal underlying molecular pathology of the secondary injury response and advance 4-AP translation to reduce axon damage and stimulate activity-dependent repair after acute TBI.

**Supplementary Information:**

The online version contains supplementary material available at 10.1186/s40478-025-02219-1.

## Introduction

Axon damage is a hallmark pathology of traumatic brain injury (TBI) that disrupts neural circuits and impairs execution of major brain functions [5, 31, 88]. During a TBI event, mechanical forces can disconnect axons from neuron cell bodies. However, the majority of axon damage occurs after the initial event and results from secondary injury processes that expand pathology to broader axon populations and/or cause the initial axon damage to progress to irreversible fragmentation [5, 55]. No FDA-approved treatments promote recovery of disconnected axons or protect intact axons against secondary injury processes in patients after TBI [13, 95]. Importantly, the acute secondary injury interval may present a therapeutic opportunity for intervention prior to irreversible axon degeneration [38, 63].

TBI secondary injury involves multiple pathological processes that can impair axon health [8, 58]. Secondary axon degeneration begins days to months after TBI and ongoing axonal pathology can extend into the chronic phase [43, 66, 67]. Microbleeds identify sites of diffuse axonal and/or vascular injury [33, 61]. Vascular trauma and blood–brain barrier (BBB) disruption result in extravasation of fibrinogen that activates innate immune cells and increases axon damage [20, 77]. The innate immune response develops as microglia and astrocytes transition to reactive disease-associated glia that, if unresolved, can fuel white matter pathology with continued axon degeneration [15, 21, 44, 96]. TBI white matter pathology involves axon damage and demyelination, i.e. damage to myelin sheaths around axons, which slows or blocks axon signal conduction [3, 5, 63, 90]. Additionally, TBI disrupts nodes of Ranvier (NoR) required for action potential propagation along myelinated axons [54, 63, 78, 88]. These and other pathological features present potential targets for therapeutic intervention.

The FDA-approved small molecule, 4-aminopyridine (4-AP; fampridine, dalfampridine), was developed to enhance axon signal conduction through demyelinated regions [9, 85]. 4-AP binds to Kv1 channels exposed by demyelination which blocks potassium efflux to prolong action potential duration, enhancing signal propagation to distal NoRs and facilitating synaptic activity [14, 23, 46, 98]. 4-AP provides symptomatic relief for walking disability in patients with multiple sclerosis (MS), yet the specific mode of action in MS is unknown [23, 57, 108]. 4-AP has shown promise in ameliorating neurological symptoms caused by Kv1.2 gain-of-function mutations in patients with KCNA2-encephalopathy [36]. Additionally, insights from human genome-wide association studies led to 4-AP testing in healthy participants and showed enhanced working memory in low-performing individuals, which was linked to increased cortical excitability [73].

4-AP clinical use in MS and our preclinical findings of demyelination and NoR disruption after concussive TBI led us to test 4-AP as a treatment for axon-myelin pathology after TBI [63, 69, 78]. Using highly sensitive and specific experimental techniques to detect axon pathology in the corpus callosum (CC), we found that acute 4-AP reduced: (a) axonal swellings and NoR disruption detected in Thy1-YFP-16 mice, and b) demyelination and axonal cytoskeleton collapse quantified with transmission electron microscopy in C57BL/6 mice [78].

This preclinical study now advances our hypothesis that 4-AP treatment can improve axon-myelin health after TBI by addressing critical gaps toward translation of acute 4-AP as a treatment for axon damage after TBI. In a non-penetrating concussive TBI mouse model, we employ multiple measures shown to identify axon damage in human tissues to demonstrate 4-AP treatment efficacy along with safety measures based on seizure susceptibility. Furthermore, unbiased genome-wide spatial transcriptomics enabled comprehensive molecular profiling to characterize the secondary injury response in the tissue pathological context and evaluate the effects of acute 4-AP treatment.

## Materials and methods

### Animals

All animal procedures were carried out under Institutional Animal Care and Use Committee (IACUC) approved protocols in accordance with the US NIH Guide for the Care and Use of Laboratory Animals with care taken to limit pain and distress. Supplementary Methods Table S1 details the animal housing, numbers, and sex. Data reporting followed the ARRIVE guidelines [76] and the common data elements for preclinical TBI research [87, 102]. No mice required exclusion during the studies.

### TBI and sham procedures

Surgical procedures were performed as previously described [69]. Briefly, under isoflurane anesthesia 8 week old mice had the skull exposed by midline incision and a controlled closed-skull impact was delivered 1.5 mm in depth onto the intact skull surface at a velocity of 4 m/s at bregma (0 mm A-P) using a pneumatic device equipped with a 3-mm rounded steel impactor tip. Sham mice underwent anesthesia and incision without impact.

### Drug administration

As previously detailed [78], 4-AP (fampridine; Sigma-Aldrich Cat#275,875) was dissolved in sterile saline (0.9% sodium chloride; Fisher Scientific, Cat# NC9054335). The primary regimen used a low 4-AP dose (0.5 mg/kg, i.p.) that achieves blood serum levels in mice of 20–100 ng/ml [78], which is the therapeutic target range for 4-AP use in MS [7, 17]. The seizure risk and 4-AP dosing study included a second 10 × higher 4-AP dose (5 mg/kg) as a threshold level of increasing 4-AP seizure risk (ED_50_ = 10.9 mg/kg)[105] for testing 4-AP safety relative to efficacy for reducing axon damage. 4-AP or vehicle i.p. injections were initiated 1 day after surgery and continued twice daily (*b.i.d*.) for 6 consecutive days, with a final injection on day 7.

### Study 1: node of Ranvier organization

Confocal images of tissues generated in a prior TBI study [78] were analyzed for additional metrics of NoR molecular organization in Thy1-YFP-16 mice (Table S1), which show axon damage swelling predominantly in the CC [63]. Mice received saline or 4-AP (0.5 mg/kg) *b.i.d.* with analysis at 7 days post-injury, as explained above. NoR domains were identified by immunolabeling for Nav1.6 voltage-gated sodium channels in nodes, contactin-associated protein (Caspr) in paranodes, and Kv1.2 voltage-gated potassium channels in juxtaparanodes. NoR quantitative analysis was conducted with blinding and randomization using ImageJ as detailed in Supplementary Methods.

### Study 2: seizure risk and 4-AP dosing

This randomized, controlled preclinical interventional trial in mice followed a predetermined study design (https://osf.io/7yvtn/) with injury and drug controls, as well as experimenter blinding during execution and data analysis. The primary outcome measure of 4-AP efficacy was axonal damage within the corpus callosum (CC) identified by β-amyloid precursor protein (β-APP) immunoreactive accumulations at 7 days post-injury. Detailed procedures for injury, dosing, seizure behavior, neuropathology, and blood biomarker analyses are provided as Supplementary Methods. C57BL/6 J mice (RRID:IMSR_Jax:000664; Jackson Laboratories) received TBI or sham procedures at 8 weeks of age as separate male or female cohorts (1:1, total n = 96, Table S1) to avoid confounds in behavior assessment. After the final injections of 4-AP (0.5 mg/kg), 4-AP (5 mg/kg) or vehicle control, mice were video recorded in individual cages for one hour. Seizure behavior was scored using the Racine scale with modifications validated for mice based on electroencephalography (EEG) [101]. For each mouse, seizure severity was defined by the maximum Racine score observed. Seizure activity was corroborated by rearing and digging measures of general mobility and exploratory behavior. An additional cohort was screened for seizure behavior at 24 h post-surgical procedures to strengthen interpretation of acute TBI seizure risk (Table S1). Cardiac blood was collected approximately one-hour after video sessions and immediately prior to perfusion. Neurofilament light chain (NfL) serum biomarker analysis was processed as previously described [107], using the single molecule array (Simoa®) HD-1 Analyzer (Quanterix). After transcardial perfusion with 4% paraformaldehyde, brains were coronally cryosectioned (14 µm) followed by immunohistochemistry and microscope image acquisition using our published protocols [63, 78] with commercial antibodies (Tables S2-3). Immunolabeling was quantified using NIH ImageJ software (RRID:SCR_003070). Damaged axons in the CC were identified by swellings with accumulated β-amyloid precursor protein (β-APP) immunolabeling [92]. Gliosis was quantified in the hippocampus as the percent area immunolabeled as astrocytic with glial fibrillary acidic protein (GFAP) or microglial with ionized calcium-binding adaptor molecule 1 (IBA1).

### Study 3: genome-wide spatial transcriptomics

Unbiased spatial transcriptomics profiling was conducted as an exploratory study to identify comprehensive molecular changes resulting from low dose 4-AP treatment in relation to TBI-induced pathology (Table S1). At 8 weeks of age, 24 male C57BL/6 J mice received TBI or sham procedures followed by 4-AP (0.5 mg/kg) or saline vehicle injections, as explained above, with analyses at 7 days post-injury. Only males were analyzed with this resource intensive approach since sex was not a significant biological variable in outcome measures of axon damage, NfL serum levels, or seizure behavior at 7 days in Study 2. Analysis of coronal tissue sections of formalin-fixed paraffin-embedded (FFPE) for transcriptomics was complemented by immunohistochemistry of adjacent sections. Full details for the following experimental procedures are provided as Supplementary Methods.

*Brain FFPE Tissue Sections*: After the last injection on day 7 post-injury, mice were perfused with 10% formalin fixative (Leica Biosystems, Cat#3,800,541) and extracted brains stored in fixative for no longer than 3 days. Dissected 3-mm tissue blocks centered with bregma (0 mm A-P) were embedded in paraffin for coronal sectioning. Sections from each mouse had RNA quality confirmed as DV200 greater than 50%, measured using the Fragment Analyzer 5200 (Agilent; Cat#M5310AA) and DNF-472 High Sensitivity RNA Kit (Agilent, Cat#DNF-472-FR). Coronal FFPE brain tissue Sects. (7 µm) were prepared for Visium CytAssist spatial transcriptomics prepared into cDNA libraries and sequenced using Illumina NovaSeq sequencers (further details in Supplemental Information).

*FFPE Immunohistochemistry*: Coronal Sects. (7 μm) anterior to the spatial transcriptomics sections and under the TBI impact site were immunolabeled for myelin basic protein (MBP), β − APP, GFAP, or IBA1 (Table S2).

*Spatial Transcriptomics Data Analysis*: Visium Spatial RNA-seq and microscope images were processed for all 24 mice using 10 × Genomics Space Ranger analysis pipeline (v2.1.1) to align raw reads to the mouse reference genome and generate gene count by spot matrices aligned to corresponding tissue images. Based on the detected UMIs and genes in each spot, quality controls (QC) were applied. Five neuroanatomical regions of interest were manually defined for each sample using the Loupe browser. Downstream analysis and visualization used the R package Seurat (v5.2.0) [91]. Clusters of spots were detected using Seurat unsupervised shared-nearest-neighbor (SNN) clustering on a cell state gene set (gene n = 264).

*Differential Expression Analysis*: The spot level spatial transcriptomics data were collapsed to 5 pseudo-bulk profiles per sample by taking the sum of expression per manually defined neuroanatomical region of interest (ROI). The pseudo-bulk dataset was then analyzed using DESeq2 (v1.48.2) [60, 97], which normalized the raw gene counts and corrects for laboratory sample batch prior to testing for differentially expressed genes.

*Spatial Mapping of Cell Gene Sets*: To spatially map mouse brain cell types defined by single-cell transcriptomics, we used cell2location (v0.1.3) [50] with Visium data. Briefly, we first estimated reference expression signatures for major cell types in healthy adult mouse brain using regularized negative binomial regressions and 2.3 million single-cell transcriptomes from The Allen Brain Cell Atlas [106]. We then calculated cell-type proportions using the cell-type-specific abundance estimations for each spot in the Visium sections with the inferred reference cell state signatures.

*Pathway Analysis and Gene set enrichment analysis (GSEA)*: GSEA was conducted using the R package fGSEA (v1.34.2) [52] to identify enriched gene sets. The analysis includes both customized, published [32, 80], and publicly available MSigDB (https://www.gsea-msigdb.org/gsea/msigdb) gene sets for cell phenotypes and molecular pathways. The fGSEA method utilizes a ranked list derived from DESeq2 outputs for differentially expressed genes (DEGs), with the ranking determined by the 'stat' column from the DESeq2 results.

### Statistical analyses

See figure legends for applied statistical tests and Supplementary Information for additional details.

*Studies 1 and 2*: Statistical analyses were conducted using GraphPad Prism v10.4.1 (RRID:SCR_002798). Statistically significant results were defined as adjusted *p*-values of < 0.05.

*Study 3*: Differential gene expression was calculated for each ROI using R package Seurat v5.2.0 (RRID:SCR_016341)[91]. Pathways enriched in these differentially expressed genes were detected using fGSEA v1.34.2 (RRID:SCR_020938) for GSEA. Statistically significant results were defined as false discovery rate adjusted *p*-values < 0.1.

## Results

### Acute 4-AP therapy reduces neuropathologic indicators of axon damage.

**Fig. 1:**
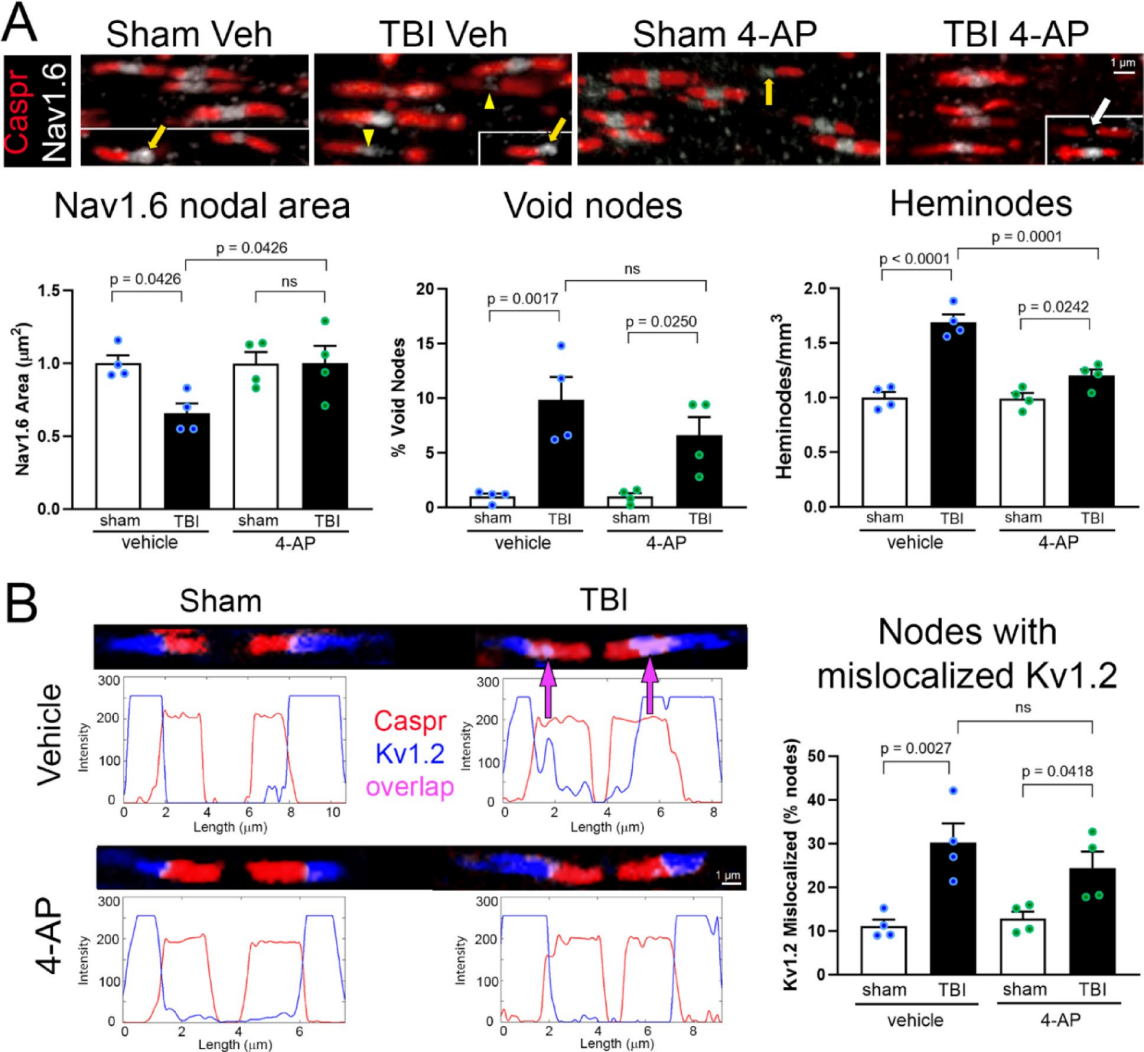
4-AP therapy reduces early-stage axon damage identified by node of Ranvier disruption after TBI. Nodes of Ranvier (NoR) are critical for myelinated axon signal conduction. TBI can disrupt the molecular organization of these highly specialized axonal domains. Confocal images of the corpus callosum (CC) illustrate axonal immunolabeling of NoR components of nodes with Nav1.6 (white in A), paranodes with Caspr (red in A and B), or juxtaparanodes with Kv1.2 potassium channels (blue in B). **A** Representative CC images show abnormal NoRs with Caspr paranode domains flanking a diminished Nav1.6 area (yellow arrowheads), or void of Nav1.6 immunolabeling in the nodal region (white arrow), or as heminodes (yellow arrows) missing a Caspr immunolabeled paranode on one side. These forms of NoR disruption are significantly increased in the CC after TBI, while 4-AP treatment improves NoR integrity. Graphs show values normalized to respective shams. **B** Representative CC images with NoR immunolabeling intensity profiles. Sham mice exhibit the normal restriction of Kv1.2 channels (blue) to the juxtaparanode domains that are adjacent to immunolabeling for Caspr (red) in paranodes. After TBI, Kv1.2 channels often spread beyond the juxtaparanode domain to overlap (magenta; arrows) with Caspr immunolabeling in the paranode domain. This Kv1.2 mislocalization is significantly increased after TBI and only partially recovers with 4-AP treatment. Bars represent mean ± SEM with an individual data point shown for each mouse. Images analyzed are from Thy1-YFP-16 mice balanced for male and female sex; sex differences were not found in our prior study of axon damage in these Thy1-YFP-16 cohorts [78]. See Supplementary Tables S1 and S4 for details of mouse numbers, sex, and statistical analysis

We demonstrate beneficial effects of acute 4-AP (days 1–7 post-injury) on axon damage in the CC after concussive TBI in mice using multiple neuropathological measures that each identify specific axon pathophysiology. First, we examined NoR integrity as an indicator of early-stage axon damage and specifically Kv1 channels, a putative 4-AP therapeutic target (Fig. 1). Changes in NoR domains and Kv1 localization (Fig. 1) were quantified using metrics reported for human neuropathology with MS and/or TBI specimens [29, 40, 88, 100]. 4-AP was administered at a low dose of 0.5 mg/kg that produces blood levels of 20–100 ng/ml within 30–60 min [78], which approximates the target therapeutic range for patients with MS [17]. Several parameters of NoR disruption were evident due to TBI in adult mice (Fig. 1). Typical NoR structure includes a central nodal region, marked by Nav1.6 sodium channel immunolabeling, that is flanked by two Caspr-immunolabeled paranode domains. TBI diminished Nav1.6 immunolabeling in nodal regions (*p* = 0.0426; Fig. 1A) and increased the proportion of axonal NoRs lacking detectable Nav1.6, *i.e.* void nodes (*p* = 0.0017; Fig. 1A). TBI also increased the presence of heminodes, which have a single Caspr domain adjacent to a nodal Nav1.6 domain rather than the normal pair of flanking Caspr paranodes (*p* < 0.0001; Fig. 1A). Furthermore, TBI disrupted Kv1.2 potassium channel localization, which was no longer restricted to the juxtaparanodal region under the myelin sheath (*p* = 0.0027; Fig. 1B). Dispersion of Kv1.2 immunolabeling into the paranode domain indicates myelin detachment that exposes Kv1.2 channels to ionic flux with the extracellular environment. TBI-induced NoR disruption was attenuated by 4-AP treatment. Low dose 4-AP (0.5 mg/kg) treatment on days 1–7 significantly improved NoR integrity based on nodal Nav1.6 area (*p* = 0.0426; Fig. 1A) and Caspr heminodes (*p* < 0.0001; Fig. 1A). The 4-AP treatment did not eliminate void nodes (Fig. 1A) or nodes with mislocalized Kv1.2 (Fig. 1B). These findings add metrics used in human TBI and MS tissues [29, 40, 88] to further our initial analysis in mice [78] to demonstrate that 4-AP ameliorates specific NoR metrics of early-stage axon damage after TBI.

**Fig. 2:**
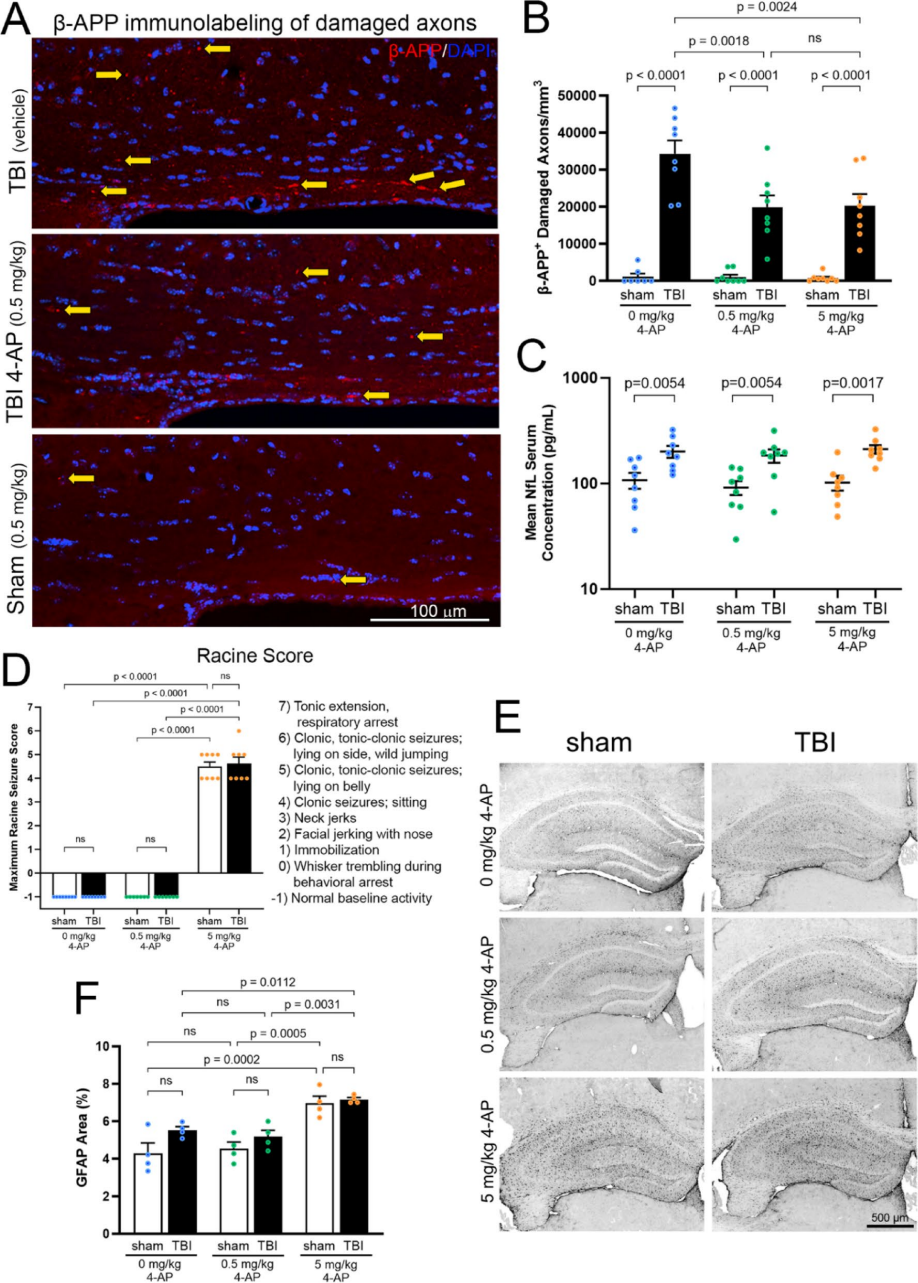
Low dose 4-AP therapy effectively reduces axon damage associated with impaired axonal transport after TBI, without increasing indicators of seizure risk. **A** β-amyloid precursor protein (β-APP) immunolabeling in coronal sections of the corpus callosum at 7 days after TBI or sham procedures. Arrows (yellow) point to examples of accumulation of β-APP immunolabeling (red), which detects axonal swellings with impaired axonal transport. Nuclei are stained with DAPI (blue). **B** The density of axonal swellings detected with β-APP immunolabeling is significantly increased after TBI and significantly reduced with 4-AP treatment on days 1–7 post-TBI. 4-AP efficacy was highly significant at both low (0.5 mg/kg) and high (5 mg/kg) doses. **C** Serum levels of neurofilament light chain (NfL) protein are significantly elevated 7 days after TBI, as compared to sham controls. Neither low nor high dose 4-AP treatment normalized NfL significantly toward sham levels at 7 days post-injury. **D** Mice were video recorded for 60 min after the final drug/vehicle injection on day 7 and scored according to the Racine scale for seizure behavior indicators. High dose of 4-AP (5 mg/kg) produced a progression of seizure behaviors, from behavioral arrest to motor effects, which confirmed scoring procedures with the Racine scale. In contrast, seizure behavior was not observed in mice with the low therapeutic dose of 4-AP (0.5 mg/kg) or in vehicle control mice. Also, TBI did not induce behavioral indicators of seizure activity. **E** Coronal sections through the hippocampus, a site susceptible to seizure activity, illustrate astrogliosis detected with glial fibrillary acidic protein (GFAP) immunolabeling. Astrogliosis is most prominent with high dose 4-AP (5 mg/kg) in both sham (left) and TBI (right) mice. **F** Hippocampal astrogliosis was significantly increased with the high 4-AP dose (5 mg/kg), but not with the low 4-AP dose (0.5 mg/kg), as compared to vehicle controls (E). Cohorts were balanced for male and female with no statistical differences found between the sexes for β-APP, NfL, or Racine scores. See Supplementary Materials Table S1 and S5 for details of mouse numbers, sex, and statistical analysis

Next, we evaluated 4-AP (days 1–7 post-injury) efficacy based on CC axon damage identified by β-APP immunohistochemistry (Fig. 2A, B), the “gold standard” for axon damage in clinical neuropathology [103, 104]. β-APP immunolabeling detects vesicles and organelle accumulations from impaired axonal transport and/or axon fragmentation. 4-AP efficacy at the low 0.5 mg/kg dose relevant to symptomatic treatment in patients with MS [78] was compared with a 10×-higher dose of 5 mg/kg, considered the potential maximum tolerable dose due to increased seizure risk (ED_50_ = 10.9 mg/kg) [105]. TBI significantly increased axonal swellings with accumulation of β-APP immunolabeling (*p* < 0.0001; Fig. 2A, B). Among injured mice, axon damage was significantly reduced with 4-AP treatment at either 0.5 mg/kg (41.9%; *p* = 0.0018) or 5 mg/kg (40.9%; *p* = 0.0024; Fig. 2A-B). These results show robust efficacy in both 4-AP groups and sufficiency of low dose 4-AP for reducing axon damage.

Finally, axon damage was estimated from serum NfL protein levels (Fig. 2C). NfL is a translationally relevant biomarker of axon microstructural damage that is predictive of functional outcomes after TBI [31, 83, 99]. NfL levels increase over time after TBI, which may reflect the extent of axon damage from evolving secondary brain injury [6, 31, 99]. We used the Simoa® NfL assay, which is effective in clinical TBI studies and in mouse TBI models [31, 107]. Peripheral blood was collected just prior to perfusion of the mice for neuropathology. TBI significantly increased NfL levels as compared to sham (Fig. 2C: vehicle, *p* = 0.0054; 4-AP 0.5 mg/kg, *p* = 0.0054; 4-AP 5 mg/kg, *p* = 0.0017). This NfL data provides additional evidence that all injury groups exhibited a similar extent of axon damage. However, 4-AP treatment did not significantly alter NfL levels at this 7-day time point, possibly indicating a required exposure interval for 4-AP treatment [78] and/or insufficient time for the full extent of NfL blood levels to be reached [31, 49, 99].

### Low dose 4-AP does not induce seizure behavior or hippocampal gliosis.

Seizure effects of 4-AP dosing were analyzed in the same mice shown for axon damage with β-APP and NfL (Fig. 2D–F). Seizure behavior was scored using a modified Racine scale for mice that has been validated relative to EEG [101]. This observational scale avoids invasive EEG confounds burr holes in the skull and placement of cortical electrodes, which causes aberrant neuronal activity that takes several days to recover [70, 101]. High dose 4-AP (5 mg/kg) induced clear seizure behavior in both sham and TBI mice that progressed from behavior arrest to motor effects without escalating to the highest seizure severity (*p* < 0.0001; Fig. 2D). Furthermore, high dose 4-AP interrupted two natural behaviors, rearing (*p* < 0.0001) and digging (*p* < 0.0001) (Fig. S1A-B). In contrast, low dose 4-AP (0.5 mg/kg) did not induce seizure behavior (Fig. 2D). On day 7, mice given 0.5 mg/kg 4-AP scored similarly to vehicle controls in Racine behaviors, digging, and rearing. Clinically, acute symptomatic seizure incidence is highest during the 7 days after TBI, particularly during the first 24 h [35, 75, 79]. The behavioral findings of this 7-day study were reproduced in an additional cohort of mice analyzed at 24 h post-injury (Table S1, Fig. S2). Therefore, TBI did not alter susceptibility to seizure behaviors.

A lack of seizure induction with low dose 4-AP is corroborated by neuropathological analysis of gliosis in the hippocampus (Fig. 2E), a brain region prone to seizure activity [19]. Astrogliosis is a defining histological feature and potentially contributes to seizure induction [74]. Seizure activity from high dose 4-AP (5 mg/kg) induced reactive astrogliosis (*p* < 0.0002; Fig. 2F) along with microgliosis (*p* = 0.0102; Fig. S1C). Importantly, low dose 4-AP (0.5 mg/kg) did not increase gliosis in the hippocampus, as compared to the vehicle control of the sham or TBI groups (Fig. 2F, S1C).

### Genome-wide spatial transcriptomics reveals TBI and 4-AP effects on disease-associated glial cell phenotypes

**Fig. 3:**
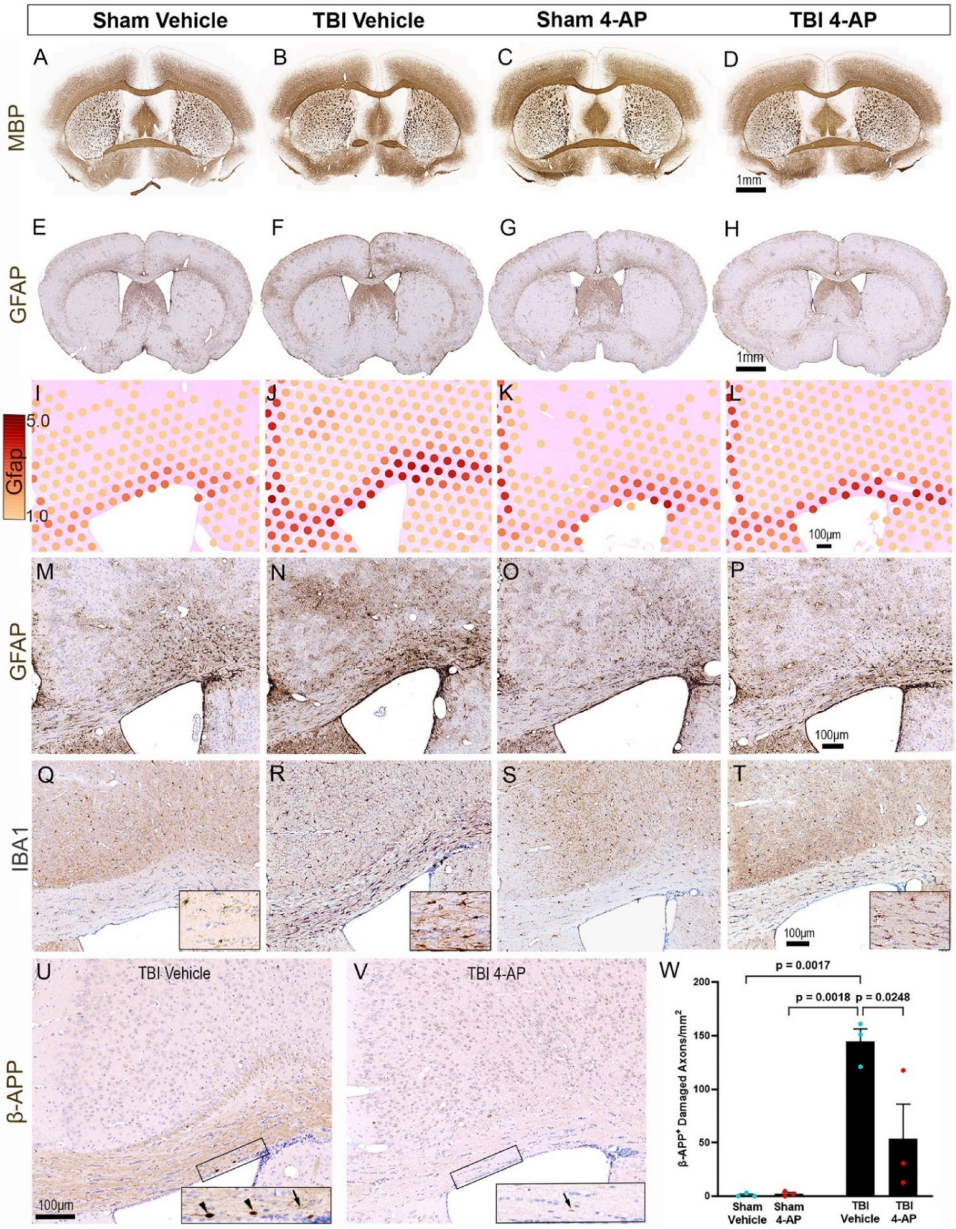
TBI-induced axon damage in the corpus callosum is associated with active gliosis and reduced by 4-AP at 7 days post-injury. Formalin-fixed paraffin-embedded (FFPE) coronal tissues aligned with the impact site were examined in each of the four mouse groups: sham or TBI with vehicle and sham or TBI with 4-AP (0.5 mg/kg). Immunohistochemistry was performed on sections adjacent to those used for spatial transcriptomics analysis. **A-D** Myelination detected with myelin basic protein (MBP) illustrates similar patterns of myelinated axons in the corpus callosum (CC) and cortical regions that project axons through the CC. TBI does not cause extensive demyelination or focal lesions. **E–H** Astrogliosis detected by strong glial fibrillary acidic protein (GFAP) immunoreactivity is prominent in the CC and cortical regions after TBI, as compared to sham controls. **I-P** Spatial transcriptomics demonstrates active astrogliosis after TBI mice as indicated by abundant *Gfap* mRNA transcript expression in the CC (I-L) that corresponds with the hypertrophic morphology of reactive GFAP immunolabeled astrocytes (M-P). **Q-T** Microgliosis detected by strong ionized calcium-binding adaptor molecule 1 (IBA1) immunoreactivity is also prominent in the CC after TBI, as compared to sham controls, and corresponds with thickened processes and rounded cell bodies of reactive microglia. **U-W** Axon damage identified by axonal profiles immunoreactivity for β-amyloid precursor protein (β-APP) increases after TBI and is significantly reduced with 4-AP treatment. One way ANOVA with Holm-Sidak correction for multiple comparisons for n = 3 male mice for each of the four groups

Genome-wide spatial transcriptomics enabled comprehensive unbiased characterization of the secondary injury response at 7 days after non-penetrating TBI, which revealed multifactorial secondary injury processes and 4-AP treatment effects. Experiments in male mice used low dose 4-AP (0.5 mg/kg) with randomization, blinding, and controls of sham injury and vehicle injections. Spatial transcriptomics captured transcripts enabled localization of gene expression relative to structural neuropathological features identified by immunohistochemistry in adjacent tissue sections. Immunolabeling for MBP in myelin sheaths illustrates the interhemispheric circuit of cortical regions projecting callosal axons through the CC white matter and demonstrates the absence of cortical cavitation or white matter focal lesions from TBI (Fig. 3A–D). Reactive astrocytes localized to the injured CC and extended into the cortical regions under the impact site as identified with GFAP immunolabeling (Fig. 3E–H) and spatial transcriptomics of *Gfap* expression (Fig. 3I–L), which also provides technique validation. GFAP expression after TBI appears attenuated by 4-AP treatment with both *Gfap* gene expression (Fig. 3I–L) and GFAP protein immunolabeling (Fig. 3M–P). IBA1 immunolabeling detected reactive microgliosis throughout the CC in response to TBI, which also appeared attenuated by 4-AP treatment (Fig. 3Q–T). Importantly, TBI increased CC axon damage (*p* = 0.0017), which 4-AP significantly reduced in these spatial transcriptomics mice (*p* = 0.0248) (Fig. 3U–W) as in the seizure and dosing study (Fig. 2A).

**Fig. 4:**
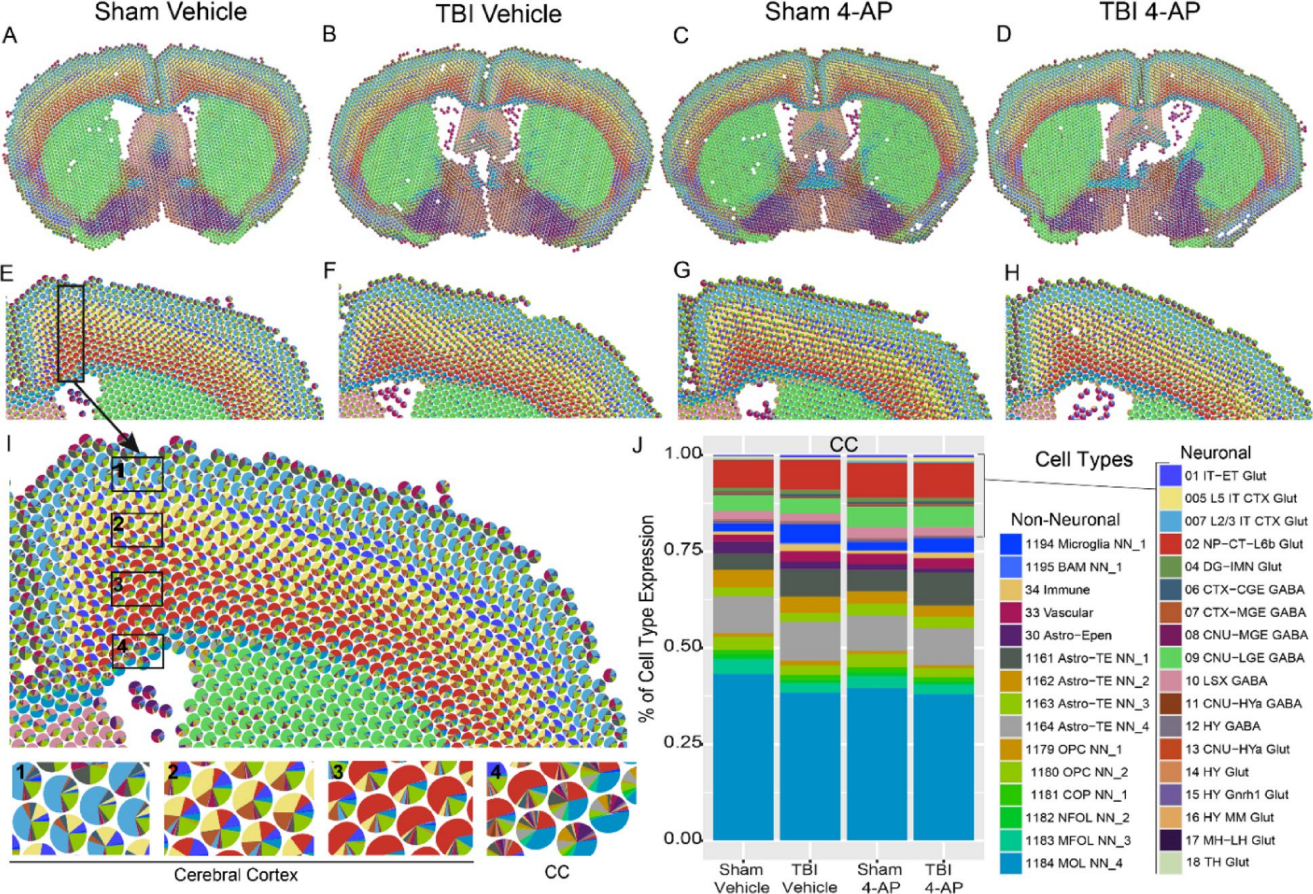
This non-penetrating TBI does not cause overt disruption of tissue cytoarchitecture or marked cellular infiltration at 7 days post-injury. Formalin-fixed paraffin-embedded (FFPE) coronal tissues aligned with the impact site were examined by spatial transcriptomics using cell2location with healthy adult mouse brain gene sets for homeostatic cell populations. Cell types were mapped onto Visium spots (55 µm) across whole brain tissue sections for mice in each of the four groups: sham or TBI with saline vehicle and sham or TBI with 4-AP (0.5 mg/kg). **A–D** Representative scatterpie charts show the proportion of cell types per spot identified by cell type-marker gene expression from cell2location mapped onto representative whole brain sections all four mouse groups. **E–H** Enlargement of cerebral cortex and subcortical white matter from A-D. TBI does not produce overt changes in the molecular signatures for cell populations across the four groups. **I** Enlargement from E illustrates cell type variation across cortical and white matter regions. **J** Quantitative analysis focused on the CC as the main site of TBI-induced axon damage and neuroinflammatory gliosis. Mature oligodendrocytes represent the largest population within the CC white matter. The proportions of annotated cell types were not significantly different across groups, based on pairwise comparisons of groups with Kolmogorov–Smirnov test

To identify cell population responses to TBI and 4-AP treatment, we annotated cell types within our spatial transcriptomics data using a reference dataset for healthy adult mouse brain [106]. Within the white matter and cerebral cortex under the impact site, homeostatic neuronal and glial molecular signatures detected in each spot were consistently region specific yet not markedly altered after injury and/or 4-AP treatment (Fig. 4).

**Fig. 5:**
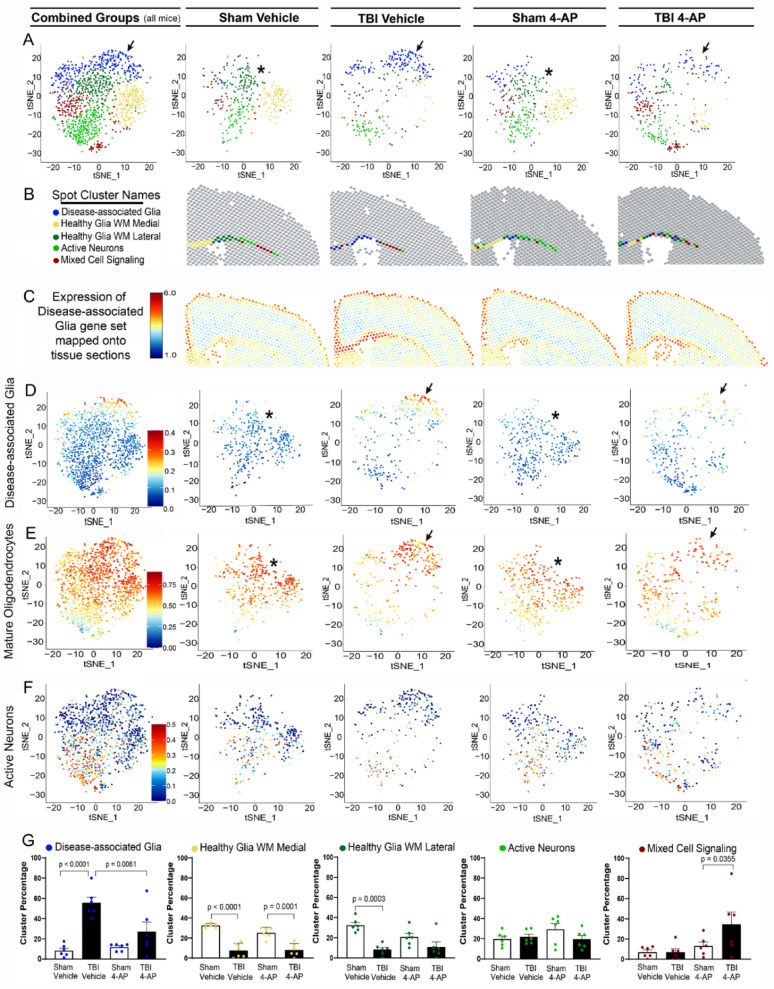
Disease-associated glia gene sets localize within the corpus callosum after TBI and are reduced with 4-AP treatment. Gene expression in Visium spots mapping to the corpus callosum (CC), the predominant site of TBI pathology, was further investigated using a restricted set of genes relevant to glial and neuronal cell states (Table S8). **A** CC spots combined from all 24 mice (sham or TBI with vehicle; sham or TBI with 0.5 mg/kg 4-AP) separated into five clusters with distinct expression patterns of the restricted gene set. The upper spot cluster (blue) is more prominent after TBI (arrows) as compared to the lack of spots distributed to this cluster from sham mice (asterisks). Each cluster molecular signature is characterized by expression of the restricted gene set and all other genes expressed within the clustered spots (Table S9). **B** Clustered spots also mapped onto the CC with distinct patterns in representative tissue section for each of the four groups. Based on the molecular signatures and localization, the five clusters are referred to as Disease-associated Glia (blue), Healthy Glia White Matter (medial: light yellow, lateral: dark green), Active Neurons (light green), and Mixed Cell Signaling (red).**C**, **D** Expression of the disease-associated glia gene set mapped onto tissue sections and spot clusters is highest after TBI in the CC and upper spot cluster (arrows). **E** Mature oligodendrocytes are the largest CC cell population (see Fig. 4J). Expression of the mature oligodendrocyte gene set indicates a subset shift to a disease-associated cell state after TBI (arrows). **F** The gene set of immediate early genes expressed in active neurons is localized in spots within the lower clusters across groups. **G** The percentages of total CC spots within each cluster show that TBI induces a significant increase of expression for the disease-associated glia cell state and reduces healthy glial cells. In injured mice, 4-AP mitigated the expression of disease-associated glial states and increased cell signaling genes. In sham mice, 4-AP did not significantly shift glial or neuronal cell states, as compared to vehicle. P-values from one way ANOVA with Holm-Sidak correction for multiple comparisons

We then further investigated cellular responses in the CC white matter site of axonal injury and 4-AP benefit. We developed a custom list of restricted genes to distinguish cell states of disease-associated glia and active neurons (Table S8 shows gene sets with supporting citations). Clustering of CC spots across all treatments revealed five clusters with distinct gene expression profiles (Fig. 5A, B; Table S9 shows genes expressed in each cluster). TBI induced expression of a disease-associated glia signature that mapped to the CC (Fig. 5C, D) and included a subset of mature oligodendrocytes (Fig. 5E), among astrocyte and microglial phenotypes (Fig. S3). Neuronal activity genes in the CC spots were not significantly altered by injury or 4-AP (Fig. 5F, G). Based on the CC spot clusters in each treatment group, the cluster representing disease-associated glia was significantly increased after TBI (*p* < 0.0001; Fig. 5G). Another cluster representing healthy glial cells, was reduced in TBI samples (*p* < 0.0001; *p* < 0.0003; Fig. 5G). 4-AP treatment significantly reduced disease-associated glial signatures that were elevated after TBI (*p* = 0.0061; Fig. 5G). Finally, in injured mice, 4-AP increased the prevalence of the cluster for mixed cell signaling genes, which could include axon-glial interactions (*p* = 0.0355; Fig. 5G).

### Differential gene expression informs TBI and 4-AP effects in the white matter and cortex

**Fig. 6:**
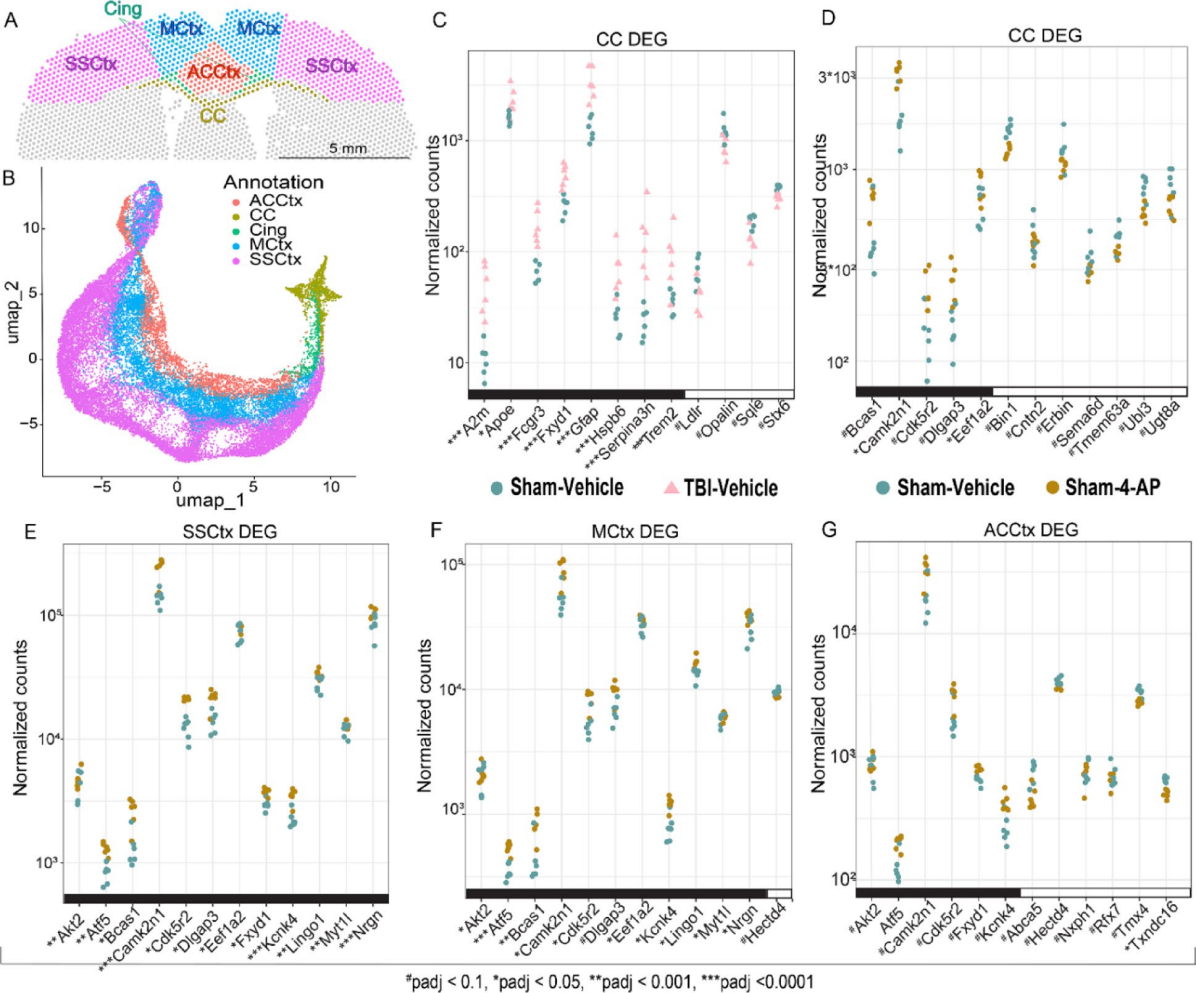
Differential gene expression (DEG) analysis in neuroanatomically defined regions. **A** Annotated region-of-interest (ROI) areas for a representative coronal brain tissue section at 7 days post-injury or sham procedure shown with the Loupe browser. The ROIs under the skull impact site include cortical regions of the anterior cingulate area (ACA), motor cortex (MCtx) and somatosensory cortex (SSCtx) along with white matter tracts of the corpus callosum (CC) and cingulum (Cing).** B** Spot expression across 5 ROIs and all mice by Uniform Manifold Approximation and Projection (UMAP) reveals that spots group together by ROI cytoarchitecture and indicates a lack of batch effects across the cohorts. **C**–**G** Selected DEGs within ROIs (each symbol represents a single mouse; n = 6 mice per condition). Genes are listed in alphabetical order and grouped for upregulation (black X-axis line) and downregulation (white X-axis line). **C** Pairwise comparison of TBI + vehicle and sham + vehicle mice groups differentiates the effect of injury in the CC, which was the predominant site of DEGs for TBI versus sham (104 DEGs). **D**– **G**Pairwise comparison of sham + 4-AP and sham + vehicle groups reveals 4-AP treatment effect on DEGs in the CC (D) and cortical ROIs (E–G) in the absence of TBI. Significance level was an adjusted *p*-value of < 0.1. *Atf5* was the DEG in the MCtx with the greatest statistical difference (*p* adj = 5.69E−06) and ranked 14th in the SSCtx (p adj = 1.42E−04) with the ACA for comparison (*p* adj = 0.101). See Supplementary Information (Table S10) for all DEG analysis across four pairwise comparisons of TBI or sham with 4-AP or vehicle

Distinct neuroanatomical regions-of-interest (ROIs) underwent pairwise comparisons to quantify differentially expressed genes (DEGs) of TBI and/or 4-AP treatment effects (Fig. 6). Expert manual review of the neuroanatomy in all H&E histological sections identified five ROIs matching with the Allen Brain Atlas [1] for the CC and corresponding motor cortex (MCtx) and somatosensory cortex (SSCtx) regions, which contain cell bodies of neurons that project callosal axons to the contralateral homotypic cortex, along with regions directly under the impact site in the anterior cingulate area (ACA) of the cortex and in the cingulum (Cing) white matter (Fig. 6A). Clustering of spot-wise gene expression shows these ROIs are distinct and consistent across mice, supporting the use of a pseudo-bulk analysis across groups (Fig. 6B). ROIs were then compared to identify statistically significant DEGs based on injury and treatment (Table S10). Canonical/known markers varied across ROIs illustrating relevant significant effects of TBI injury and 4-AP treatments. (Fig. 6C–G; see Supplementary Information for full set of DEGs in all ROIs).

TBI-induced DEGs were most prominent in the CC, based on comparing TBI + vehicle and sham + vehicle groups (Fig. 6C, Table S10). TBI increased immune and inflammatory responses and associated cellular stress genes (e.g. *A2m*, *Fcgr3*, *Gfap*, *Hspb6, SerpinA3n, Trem2*)(Fig. 6C) along with upregulation of *C4b* and *C1qa-c* complement components (Table S10). TBI also increased *Fxyd1* encoding phospholemman protein that controls cell excitability by modulating sodium–potassium balance through Na,K-ATPase pump activity [22, 65]. TBI downregulated genes related to oligodendrocytes (*Opalin*), lipid synthesis (*Sqle*), and cholesterol homeostasis (*ApoE, Ldlr*). Syntaxin 6 (*Stx6*), which mediates tau secretion from cells [56], was also downregulated. Strikingly, many TBI-induced DEGs in the CC at this acute stage are associated with neurodegenerative conditions, such as Alzheimer’s disease [16, 48] (Fig. 6C, Table S10). In comparison to the CC, the cingulum white matter tract, which runs above and rostrocaudally perpendicular to the CC, had a milder injury response with mainly downregulated expression of genes related to oligodendrocytes and myelin, including *Opalin* and a series of myelin-specific genes (*Cnp, Mag, Mal, Mobp, Mog,* and *Plp1*)(Table S10). Therefore, the DEG profiles are distinct between these white matter tracts; the secondary injury effects are more robust in the interhemispheric CC, possibly due to TBI-induced mechanical strain across the midline.

4-AP treatment alone produced broad DEG effects in the CC and cortical regions, based on comparing sham + 4-AP and sham + vehicle groups. A subset of DEGs illustrates effects of 4-AP treatment across ROIs (Fig. 6E–G, Table S10). Several increased DEGs encode key proteins (*Camk2n1*, *Cdk5r2*, *Dlgap3*, *Eef1a2*) for functions related to the cytoskeleton, protein synthesis, and synaptic plasticity. In cortical regions, increased ATF5 may regulate glial differentiation and protect neurons in response to ischemic cell stress [4, 42]. 4-AP increased DEGs related to oligodendrocyte lineage differentiation (*Bcas1*, *Lingo1, Myt1L*). However, expression decreased specifically in the CC for genes related to myelination and cholesterol (*Abca5*, *Erbin*, *Tmem63a*, *Utg8a*). 4-AP also decreased *Bin1* and *Cntn2* which regulate trafficking of receptors and ion channels. Additionally, increased *Kcnk4* in all three cortical regions with 4-AP treatment is interesting as a potential compensatory response to stabilize Kv1 channel potassium efflux. *Kcnk4* encodes K2P4.1/TRAAK channels that are localized within the NoR and axon initial segment [25].

### Secondary TBI processes and 4-AP treatment alter molecular pathways of white matter injury and cortical regions

**Fig. 7:**
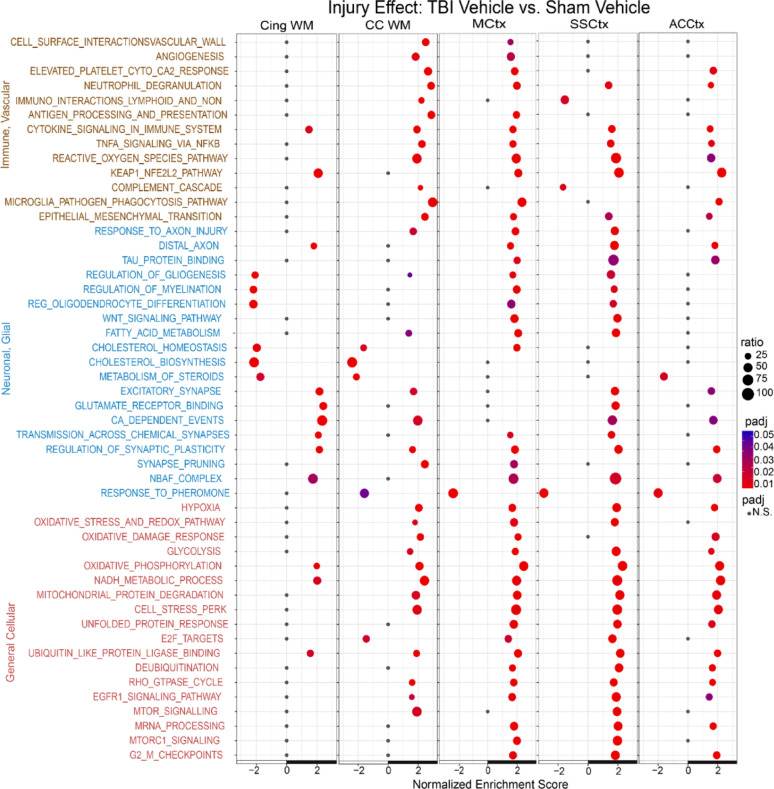
Gene set enrichment analysis (GSEA) captures the complex tissue response to TBI. Pairwise comparison of molecular pathway normalized enrichments scores between TBI or sham mice receiving saline vehicle injections with tissue collection on day 7. Pseudo-bulk ROI profiles for TBI + vehicle (n = 6) relative to sham + vehicle (n = 6) male mice. See Supplementary Information for details of the full set of significant pathways and leading-edge genes (Table S11)

**Fig. 8:**
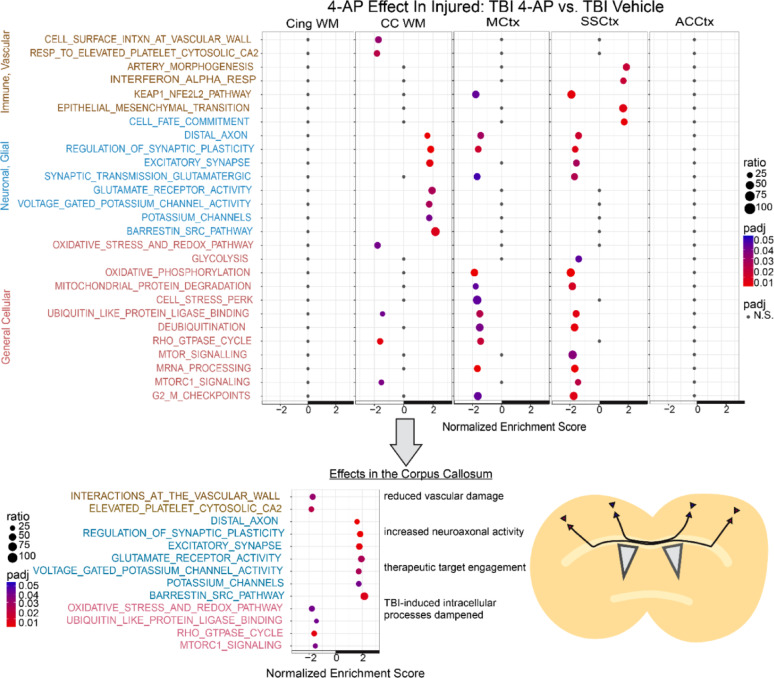
Gene set enrichment analysis (GSEA) identifies 4-AP treatment effects on the tissue response to TBI. Pairwise comparison of molecular pathway normalized enrichment scores between TBI mice receiving 4-AP (0.5 mg/kg) or saline vehicle injections with tissue collection on day 7. Pseudo-bulk ROI profiles for TBI + 4-AP (n = 6) relative to TBI + vehicle (n = 6) male mice. See Supplementary Information for details of the full set of significant pathways and leading-edge genes (Table S11)

Within the five ROIs. GSEA pathway analysis identified many significant molecular pathways relative to TBI pathology and 4-AP treatment. Pairwise comparisons differentiated effects of TBI with and without 4-AP treatment, along with injury and drug controls. Review of the molecular pathways identified many mechanistic insights relevant to this study (Fig. 7 and 8, S4-S5), among the large number that were statistically significant (see Supplementary Information, Table S11).

Comparison of TBI + vehicle and sham + vehicle groups identified significant TBI injury effects on gene expression involving molecular pathways of immune and vascular functions, neuron and glial cell responses, and general cellular processes (Fig. 7). GSEA detected vascular pathology, indicated by neutrophil degranulation [82], even in this non-penetrating model, which differed clearly from the strong peripheral cell response in a penetrating TBI model [53, 72]. TBI also increased genes for release of pro-inflammatory mediators, generation of reactive oxygen species, and activation of the KEAP1-NFE2L2 oxidative stress pathway. A neuroinflammatory response developed with complement cascade activation (*C4b, C1qc, C3ar1, C1qa, C1qb, C3*) and microglial transition to a reactive phagocytic state. Consistent with axon damage (Fig. 2 and 3), GSEA changes indicated a response to axon injury in the CC and in MCTx and SSCtx sites that project CC axons. This MCtx and SSCtx axonal injury response involves the *Sarm1* molecular pathway, which executes distal axon degeneration [12]. Intriguingly, increased regulation of myelination and glial cells in MCtx and SSCtx regions contrasted with decrease of these pathways in the cingulum. However, both cingulum and CC white matter tracts showed decreases in multiple pathways associated with lipid metabolism and biosynthesis of cholesterol, a major component of myelin sheaths, in agreement with lipidomic analysis in a penetrating TBI model [26].

All five ROIs had increased transcriptomic signatures of synaptic plasticity after TBI (Fig. 7) with most also increasing calcium-dependent events and neuronal activity, as indicated by the NBAF complex pathway [18] and supported by increased oxidative phosphorylation [59]. In the cortical and CC ROIs, TBI disruption of cellular homeostasis was evident with activation of gene expression signatures of hypoxia along with oxidative, metabolic, and bioenergetic processes. Furthermore, induction of the PERK pathway, unfolded protein response, and ubiquitin pathways indicated disrupted protein homeostasis. TBI induced multiple cellular pathways that serve diverse roles in neurons and glia. Rho GTPase activity regulates actin and microtubule proteins, including roles in synaptic plasticity and myelination. EGFR1 and mTOR with mTORC1 respond to injury and support repair process, which may be complemented in cortical regions by increased mRNA processing and the cell cycle checkpoint for G2/M phase transition. Overall, secondary injury effects in cortical regions, particularly the MCtx and SSCtx, reflected the CC white matter injury.

Comparison of TBI + 4-AP with TBI + vehicle groups identified significant 4-AP effects after injury (Fig. 8). 4-AP treatment significantly reduced pathways associated with vascular damage and oxidative stress in the CC. In this injury context, 4-AP significantly reduced the MCtx and SSCtx activation of pathways for KEAP1-NFE2L2 oxidative stress and dampened inflammatory signaling while preserving cellular homeostatic pathways. Also, in the MCTx and SSCtx, 4-AP reduced pathways associated with synaptic plasticity and excitatory synapse transmission through glutamate receptors, which may involve the distal axon projection to presynaptic terminals that synapse onto neurons in the contralateral homotypic cortex. In contrast, in the CC, 4-AP increased synaptic plasticity along with excitatory synapses and glutamate receptor activity, which include leading-edge genes associated with white matter axon-oligodendrocyte synapses and activity-dependent myelination [11, 28, 64]. Importantly, increased voltage-gated potassium channel activity was found only in the CC, which may indicate a localized allostasis response to 4-AP inhibition in regions in axons vulnerable to TBI-induced damage. The leading gene was *Kcnq2*, encoding the Kv7.2 channel, followed by a series of *Kcn* family genes for voltage-gated potassium channels that respond to changes in voltage and potassium concentrations to stabilize the resting membrane potential and NoR excitability [47].

4-AP also has potential effects in healthy adults, as was shown in a clinical trial of memory performance [73]. We also identified effects of 4-AP in the comparison of sham + 4-AP and sham + vehicle groups (Fig. S4). In the CC, 4-AP decreased oligodendrocyte differentiation and myelin assembly gene sets yet increased pathways for synaptic plasticity, excitatory synapses, and glutamate receptor activity. The pathway of ionotropic glutamate receptor binding identified leading edge genes (*Shank3, Grin1, Dlg4*) within the CC that encode an axon-oligodendrocyte synapse implicated in the connection of myelination with autism spectrum disorder pathology [28]. The absence of TBI pathology enabled detection of broader effects of 4-AP involving ion channels, including voltage-gated potassium, sodium, and calcium channels and calcium-dependent signaling events. 4-AP effects extended beyond the CC to MCtx and SSCtx for pathways involving voltage-gated and G protein-gated potassium channels along with the β-arrestin sarcoma (src) pathway, which is a key signaling cascade initiated by G protein-coupled receptors that include metabotropic glutamate receptors. 4-AP changes in cellular homeostasis pathways were evident in the CC, yet more involved in the MCtx and further in the SSCtx, potentially reflecting increased neuroaxonal excitability.

A final analysis compared TBI + 4-AP and sham + 4-AP groups (Fig. S5). Similar TBI effects in the CC were significant in this GSEA (Fig. S5) as in the above TBI + vehicle and sham + vehicle comparison (Fig. 7). Molecular pathways were increased for immune and vascular processes along with hypoxia/oxidative damage, indicating that responses to TBI are distinct from those induced by 4-AP treatment. Furthermore, 4-AP dampened TBI-induced changes in cellular homeostasis pathways (Fig. S5).

Overall, these pathway enrichment analyses identify predominant molecular responses to 4-AP treatment in the CC and in MCTx and SSCtx cortical regions of callosal projection neurons after TBI. Importantly, the combination of neuropathological and spatial transcriptomics results demonstrates that 4-AP treatment reduces axon damage, enhances molecular pathways for neuroaxonal activity, and dampens pathological processes in the CC after TBI.

## Discussion

This preclinical study advances translation of acute 4-AP to treat axon damage after TBI. We present clinically relevant measures of axon damage combined with unbiased comprehensive molecular profiling of the secondary injury response to non-penetrating TBI and demonstrate efficacy and safety metrics of 4-AP treatment. Axon damage was analyzed in the CC white matter, a clinically relevant site of diffuse axonal and/or microvascular traumatic injury [30, 31, 61]. 4-AP treatment reduced NoR disruption (Fig. 1), which is a therapeutic target and represents an early stage of axon damage in experimental TBI and human postmortem acute TBI [63, 78, 88]. 4-AP treatment reduced axon damage, using the clinical standard of β-APP immunolabeling, with dosing that did not increase indicators of seizure risk (Fig. 2 and 3). Genome-wide spatial transcriptomics localized disease-associated glial cell phenotypes in the CC (Figs. 3, 4 and 5) and revealed molecular signatures of vascular injury, immune activation, and cell stress, which contrasted with downregulated processes associated with oligodendrocytes and myelination (Figs. 6 and 7). 4-AP treatment reduced disease-associated glia signatures and induced gene expression indicating recovery of blood vessel integrity along with cellular homeostasis and myelin-related pathways as well as expression of ion channels that regulate membrane potential, and neuronal activity (Fig. 8).

The non-penetrating TBI used in these acute studies is clinically relevant to traumatic axonal injury and secondary injury processes that impact late phase neurodegeneration. The single impact onto the closed skull avoids tissue cavitation and hemorrhages to focus on traumatic axonal injury [69, 92]. The resulting pattern of damaged axons dispersed among intact axons is characteristic of diffuse axonal injury in the CC and other white matter regions of human post-mortem TBI cases [44, 63, 86, 92]. Furthermore, CC electrophysiological deficits of slowed conduction velocity corresponded with electron microscopic evidence of diffuse demyelination and NoR disruption after TBI while axon degeneration corresponded with a diminished compound action potential [63, 69, 78]. Additionally, we validated this TBI model for evaluating axon damage intervention using a genetic proof-of-concept for the SARM1 molecular pathway that executes distal axon degeneration. TBI in *Sarm1 −/−* mice significantly reduced CC axon damage, neuroinflammation, and atrophy as compared to *Sarm1*+*/*+ mice [12, 62].

Our transcriptomics data provides novel characterization of the cellular responses of secondary injury in non-penetrating TBI. Our full set of transcriptomics data will be made available as a resource for future investigations for TBI studies and/or neurological applications of 4-AP treatment. Spatial transcriptomics distinguished localized responses, *e.g.* CC versus cingulum, and enabled integrated analysis of the interhemispheric circuit formed by the CC, MCTx and SSCtx. Homeostatic cell types (Fig. 4) annotated using healthy adult mouse brain reference data [106] do not capture reactive glial cell responses to injury [34]. Expanding the healthy reference set with customized gene sets for disease-associated glia identified clear shifts of glial molecular signatures within spot clusters and ROIs that would otherwise have gone undetected (Fig. 5, S3; Tables S8-S9). Furthermore, disease-associated glia gene expression mapped predominantly onto the CC after TBI (Fig. 5), which strongly illustrates the value of spatial transcriptomics for examining CC white matter that often has not been included in TBI transcriptomics studies [41, 94]. These disease-associated glial populations include gene expression signatures for reactive astrocytes and microglia along with oligodendrocyte lineage cells, which can exhibit dynamic changes at acute and chronic stages in mouse TBI models and in human specimens [2, 81, 84, 92]. Importantly, 4-AP treatment mitigated the shift of glial phenotypes to the disease-associated cluster in injured mice (Fig. 5).

4-AP may enhance signal conduction by inhibiting Kv1 voltage-gated potassium channels located in the axon initial segment, juxtaparanode domains, and/or presynaptic terminals [14, 25]. We previously demonstrated in this TBI model that low dose 4-AP treatment increases CC axon intrinsic excitability at 7 days post-injury [78]. Our spatial transcriptomics findings indicate 4-AP target engagement in the CC after TBI increases molecular pathways associated with the distal axon, neuroaxonal activity, glutamate receptor activity, and voltage-gated potassium channel activity (Fig. 8). The leading potassium channel gene *Kcnq2* encodes Kv7.2 channels, which contribute to slow nodal potassium current that stabilizes the resting membrane potential [25]. Additionally, without TBI, 4-AP effects were more readily detected in the MCtx and SSCtx and identified increased expression of *Kcnk4* for voltage-independent K2P4.1/TRAAK potassium channels (Fig. 6, S4), which also stabilize the resting membrane potential [25]. Together, these findings reveal low dose 4-AP inhibition of Kv1 channels modulates gene expression of complementary potassium channels with functions that stabilize the resting cell membrane potential to support continued axon and neuron activity. Furthermore, 4-AP treatment modulated gene sets associated with axon-oligodendrocyte synapses and activity-dependent elongation of myelin [11, 28, 64], which supports NoR integrity and overall myelinated axon health.

Computational modeling of 4-AP effects on potassium currents relative to the excitation/inhibition balance of cortical neurons offers insights into the distinct effects of 4-AP at low and high doses [39]. Therapeutic effects of low dose 4-AP were hypothesized to produce moderate downregulation of potassium repolarizing currents, resulting in broadening of action potentials which improves signal propagation and increases synaptic coupling [39]. This model may explain symptomatic effects in patients with MS in the presence of low 4-AP levels. Additionally, this computational model may support 4-AP therapeutic effects on myelinated axon integrity in our current data and prior acute TBI study [78] and after peripheral nerve injury [98]. Low dose 4-AP enhanced neuroaxonal signaling that may engage activity-dependent mechanisms to modify function-regulating components of myelinated axons [51, 93]. In contrast, high dose 4-AP producing strong downregulation of potassium current was hypothesized to cause depolarization block that inactivates fast-spiking layer V interneurons which then fail to inhibit excitatory pyramidal neurons, creating pathways to the emergence of seizure-like dynamics [39].

Several limitations are relevant to interpreting these findings. From a technical standpoint, each 55 μm spot on the Visium platform integrates signals from a heterogeneous cell population. Thus, the molecular signatures represent mixed environments and should not be interpreted as individual single-cell data. From a translational perspective, this study of non-penetrating TBI focuses on axon damage and is not intended to fully replicate the injury pathophysiology or heterogeneity of patients who experience TBI. This study is also limited to the acute phase when axon damage is highest with β-APP immunolabeling [69, 92, 107]. The 4-AP treatment *b.i.d*. and target blood levels achieved in mice align with the dosing prescribed for patients with MS [7, 17, 78], but the study drug was not the extended release oral dalfampridine formulation. Thus, clinical studies are required to extend the current findings to patient populations.

## Conclusions

This analysis of 4-AP as an acute treatment for TBI focused on long axons in white matter tracts, which are particularly vulnerable to primary and secondary damage in TBI [10, 45, 66, 68, 86]. 4-AP reduced NoR disruption and β-APP immunolabeling, which are early features of axon damage [29, 71, 88, 89]. Therefore, 4-AP holds promise for ameliorating initial axon damage processes and interrupting molecular pathways that lead to irreversible axon degeneration [24, 27, 37]. Our transcriptomics findings reveal increased expression of potassium channels that modulate ion flux and membrane potential relative to 4-AP blockage of Kv1 channels. 4-AP also reduced disease-associated glia signatures and upregulated gene expression of synaptic plasticity and neuroaxonal activity pathways that support axon integrity and myelination. Thus, 4-AP treatment produces multifactorial therapeutic benefits to sustain axon health and activity-dependent processes after injury. As 4-AP is FDA approved for multiple CNS indications, repurposing represents a potential pathway for rapid translation to TBI as a new clinical indication.

## Supplementary Information

Additional file1 (DOCX 3038 kb)

Additional file2 (XLSX 13 kb)

Additional file3 (XLSX 2975 kb)

Additional file4 (XLSX 8187 kb)

Additional file5 (XLSX 2070 kb)

## Data Availability

Studies 1 and 2 were designed as interventional randomized, controlled preclinical trials and so followed processes aligned with clinical trials. Predetermined study designs are posted in Open Science Framework ([https://osf.io/mnrfk/files/qr867] (https:/osf.io/mnrfk/files/qr867) and [https://osf.io/7yvtn/] (https:/osf.io/7yvtn)) along with standard operating procedures for the experiments as performed. Study 3 was designed as an exploratory study to gain from the unbiased, comprehensive data generated with spatial transcriptomics. Transcriptomics analysis data are accessible in FigShare (see Supplementary Information). Visium sequencing data will be deposited in the National Center for Biotechnology Information Gene Expression Omnibus. Additional sharing of data is possible upon reasonable request. No data was used from publicly available data of other studies.
