# Supplementary material for: Mapping the secondary response to traumatic brain injury using spatial transcriptomics shows acute 4-aminopyridine treatment mitigates axonal and molecular pathology
Source: Acta Neuropathol Commun. 2026 Jan 8;14:36. doi: 10.1186/s40478-025-02219-1 (PMC12882205; doi:10.1186/s40478-025-02219-1)
Supplement: Supplementary file 1 — Additional file1 (DOCX 3038 kb) [file 40478_2025_2219_MOESM1_ESM.docx]

**SUPPLEMENTARY INFORMATION**

**4-Aminopyridine Reduces Axon Damage and Modifies Molecular Pathways**

**of Acute Traumatic Brain Injury**

Genevieve M. Sullivan, Kryslaine L. Radomski, Shaoqiu He, Matthew D. Wilkerson, Clifton L. Dalgard, Camilla Alba, Xiaomei Zi, Martin L. Doughty, and Regina C. Armstrong

**List of Items in Supplementary Information**

**Supplementary Methods**

Study Design

Mouse Husbandry

Study 1

Study 2

Study 3

**Supplementary Figures**

Figure S1. Effects of 4-AP treatment post-TBI on digging and rearing behaviors and hippocampal microglial activation at 7 days post-injury.

Figure S2. Supplemental seizure study at 1 day post-injury.

Figure S3. Healthy glia within custom gene set clusters with injury and/or drug treatment.

Figure S4. GSEA graph of pairwise comparison of sham+4-AP and sham+vehicle groups.

Figure S5. GSEA graph of pairwise comparison of TBI+vehicle and sham+vehicle groups.

**Supplementary Tables**

**Table S1**. Summary of animal group allocation across studies.

**Table S2**. List of primary antibodies used for immunohistochemistry.

**Table S3**. List of secondary antibodies used for immunohistochemistry performed in-house.

**Table S4**. Summary of statistical analyses associated with Figure 1.

**Table S5**. Summary of statistical analyses associated with Figure 2.

**Table S6**. Summary of statistical analyses associated with Supplementary Figure S1.

**Table S7**. Summary of statistical analyses associated with Supplementary Figure S2.

**Table S8**. Custom gene sets for disease-associated glia and active neurons, with citations.

**Table S9**. Differentially expressed genes among clusters derived from custom gene sets

**Table S10**. Differentially expressed genes from each pairwise comparison of groups with injury and/or drug treatment.

**Table S11**. GSEA from each pairwise comparison of groups with injury and/or drug treatment.

**Tables S8-S9** are XLS files that are also accessible in FigShare (doi 10.6084/m9.figshare.30401782)

**SUPPLEMENTARY METHODS**

**Study Design**

These preclinical studies were designed to evaluate the effects of 4-aminopyridine (4-AP) treatment in both concussive traumatic brain injury (TBI) and healthy conditions in mice, using a randomized, controlled trial design with TBI and sham injury controls assigned to either vehicle control or 4-AP treatment arms. Prior to surgery, adult mice were ear tagged, then randomized using Microsoft Excel (RRID:SCR_016137) to surgical and treatment groups. Investigators involved in administering treatments and conducting assessments were blinded to animal group allocation through the completion of data analysis using concealed unique identifiers.

TBI and sham surgical procedures are explained in the main manuscript Methods. TBI and sham mice were randomly assigned and equally distributed across treatment groups to receive either vehicle saline or low dose 4-AP (0.5 mg/kg). For the seizure risk and dosing study, a 10x higher dose of 4-AP (5 mg/kg) was added as a treatment group. One day after surgical procedures was selected as a clinically relevant interval for diagnosis and treatment initiation. Intraperitoneal (i.p.) injections started on day 1 after sham or TBI procedures continued twice daily (*b.i.d*.) about 11-13 hours apart for 6 consecutive days, with a final morning injection on day 7. The primary outcome measure of 4-AP efficacy was axonal damage within the corpus callosum (CC) identified by β-amyloid precursor protein (β-APP) immunoreactive accumulations at 7 days post-injury. The sample size for detecting the primary outcome measure was estimated based on previously published data of axon damage data with 4-AP treatment post-TBI [1] using a power of 0.8 and α level of 0.05. The number of mice is shown by sex for each study in Supplementary **Table S1**.

**Mouse Husbandry**

Upon arrival, mice were allowed to acclimate for at least 3 days before use in experiments. Mice were housed in groups of 3-5 animals per cage (27 cm x 16.5 cm x 12 cm) with enrichment under standard conditions (21-25 °C, 12:12 h light-dark cycle) and free access to standard food and water (except during behavioral testing). All experimental procedures were conducted during the light phase of the cycle. Body weight was recorded on the day of surgery (baseline measurement) and either at 1-day or 7-days post-surgery (depending on experimental endpoint) as a measure of general health. Animals were monitored daily by veterinary staff and laboratory personnel and remained in good health for the duration of the study.

**Study 1: Node of Ranvier Organization**

4-AP Treatment Regimen

Node of Ranvier (NoR) analysis did not use any newly generated mice. This new quantitative analysis was conducted from images of tissues that were acquired in a previously published study with male and female Thy1-YFP-16 (Supplementary **Table S1**). Thy1-YFP-16 fluorescent reporter mice (B6.Cg-Tg(Thy1-YFP)16Jrs/J; Jackson Laboratories; RRID:IMSR_JAX:003709;) have the neuronal Thy1.2 promoter driving yellow fluorescent protein (YFP) expression in CC axons. Sham and TBI Thy1-YFP-16 mice received either vehicle or low dose (0.5 mg/kg) 4-AP for 6 consecutive days (*b.i.d.*, i.p.) starting one day after surgical procedures and ending with a final injection and perfusion on day 7 [1].

Confocal Image Acquisition

Confocal imaging was performed using a Zeiss LSM 700 confocal microscope (RRID:SCR_017377) with a 63x/1.4 oil Plan-Apochromat objective. All images were captured using the same microscope filters and settings, and image acquisition parameters were kept consistent across all samples. Images were acquired for z-stacks of axonal YFP with paranodal contactin-associated protein (Caspr) and nodal sodium channels (Nav1.6) or as z-stacks of axonal YFP with paranodal Caspr and juxtaparanodal Kv1.2 potassium channels. For analysis of Caspr and Nav1.6, z-stack images were acquired at 0.45 µm intervals to generate an optical image stack of 68 μm × 68 μm × 5 μm. For analysis of Kv1.2 and Caspr, the acquired image size was 127 μm × 47 μm × 11 μm at 1 µm intervals. The entire field of view served as the ROI for quantification in equivalent areas of the medial CC situated above the lateral ventricle (one image per brain hemisphere per animal).

Immunofluorescence Analyses of NoR Domain Organization

Blinded and randomized analysis was conducted with ImageJ software (RRID:SCR_003070). The yellow fluorescent protein (YFP) channel was omitted from the z-stack images of immunostained tissue sections during quantification to avoid potential bias from recognizing damaged axons with prominent YFP swellings [1]. After completion of quantification, the z-stack images were reassessed to confirm that each Caspr domain counted was along a YFP axon.

Four measures of immunolabeled NoR domains were quantified: nodal Nav1.6 area, void nodes lacking detectable Nav1.6, unpaired Caspr paranodes (i.e. heminodes), and mislocalization of juxtaparanodal Kv1.2 into Caspr domains.

A. To quantify the area of nodal Nav1.6, 50 paired Caspr-immunolabeled paranodal domains flanking nodal Nav1.6 immunolabelling were randomly selected. These 50 nodal regions were outlined to measure the Nav1.6 area and the average area was calculated using the ImageJ software. Subsequently, the average area of Nav1.6 domains from the left and right hemisphere CC images were combined to obtain a single value per animal, which was then normalized to the average of the area in sham mice.

B. For quantification of void nodes (i.e., NoR without Nav1.6 immunoreactivity between paired Caspr domains [2]), 200 Caspr paranodal pairs were randomly selected per animal (100 per brain hemisphere) to determine the percentage of void nodes. Group averages were normalized to the average number of void nodes in sham mice.

C. The total number of heminodes (unpaired Caspr immunolabeled paranode) was manually counted within each acquired z-stack confocal image (2 per mouse, 1 per hemisphere). The average density of heminodes per area quantified was then normalized to the averaged values in the corresponding sham group.

D. In healthy CC axons, Kv1.2 immunolabeling localizes to paired juxtaparanodal axonal regions flanking Caspr immunolabeled paranodes. The intensity plot profile tool in ImageJ distinguished normal domain separation from mislocalized Kv1.2 immunolabeling dispersion into the Caspr-immunolabeled paranode domain. The percentage of mislocalized Kv1.2 domains was calculated out of 60 to 200 immunoreactive callosal axons per image, with group totals normalized to averaged sham control values.

**Study 2: Seizure Risk and 4-AP Dosing**

4-AP Treatment Regimen

This randomized, controlled study with blinding was conducted in male and female C57BL/6J mice (Jackson Laboratories; RRID:IMSR_Jax:000664) (**Table S1**). TBI and sham mice received either vehicle (0 mg/kg), low dose (0.5 mg/kg), or high dose (5 mg/kg) 4-AP for 6 consecutive days (*b.i.d.*, i.p.) starting one day after surgical procedures and ending with a final injection on day 7. Given that both TBI and 4-AP carry a risk for seizures, mice were video recorded immediately after the last i.p. injection on day 7 to quantify seizure behavior as measure of 4-AP safety. After the final injection on day 7, mice were individually housed in a fresh transparent cage containing standard bedding but no enrichment, food, or water. Video recordings of mouse behaviors were conducted using Noldus Media Recorder (Noldus, RRID:SCR_004074), with a side-mounted camera to capture mouse facial expressions and other behaviors accurately. After the completion of video recording, mice were deeply anesthetized for collection of cardiac blood and perfusion with 4% paraformaldehyde.

Scoring of Seizure Behaviors

Seizure behavior was quantified using the Racine scale, as modified for mice and validated by EEG [3], to identify behavioral signs of seizures resulting from either experimental TBI, drug administration, or both. The highest stage observed was recorded as the animal’s final score (i.e., scoring was not cumulative). Seizure stages were scored according to the following scale:

Stage -1, normal exploratory activity;

Stage 0, whisker trembling during immobility;

Stage 1, sudden behavioral arrest or motionless staring (immobilization);

Stage 2, facial jerking expressed with the nose;

Stage 3, neck jerks;

Stage 4, clonic seizures in a sitting position (forelimb jerking movements);

Stage 5, clonic, tonic-clonic seizures lying on belly;

Stage 6, clonic, tonic-clonic seizures lying on side and wild jumping;

Stage 7, tonic extension (of either forelimbs alone or both fore- and hindlimbs) possibly leading to respiratory arrest and death.

Rearing and Digging Behavior

As seizures can incapacitate animals, natural cage exploratory behaviors were also evaluated in the recorded videos. Within the one-hour time frame, two exploratory behaviors were quantified: 1) rearing, defined as vertical standing on hindlimbs with or without forepaw contact with cage walls, and 2) digging, defined as displacement of bedding material using the forepaw and snout. The total number of rearing and digging events for each mouse was scored by a blinded observer. These measures were analyzed as secondary indices of seizure-related behavioral suppression. Seizure activity limited the animals’ ability to perform these normal healthy exploratory behaviors.

Blood Biomarker Analysis

Cardiac blood was collected one hour after the last (or only) i.p. injection immediately prior to transcardial perfusion. Following the completion of video recording for seizure behavior evaluation, mice were anesthetized using a combination of ketamine (90 mg/kg) and xylazine (10 mg/kg). Subsequently, the chest cavity was opened, and blood was drawn from the right atrium. The collected blood samples were left at room temperature for 1-2 hours to allow clotting, followed by centrifugation at 3000 g for 10 minutes at RT to separate the serum. Aliquots were stored at −20°C until use.

Neurofilament light chain (NfL) serum levels were quantified using the single molecule array (Simoa®) HD-1 Analyzer (Quanterix Corp.) according to the manufacturer’s protocol and recommended dilution strategies. The serum concentrations of NfL were determined using Simoa® NF-light V2 Advantage reagent kits (Quanterix, Cat#104073) with a lower limit of detection at 0.085 pg/mL. All intra- and inter-plate coefficient of variation values were less than 10%. Quanterix software was used for assay data analysis. Outlier exclusion was tested prior to statistical analyses using the Robust Regression and Outlier Removal (ROUT) method with a Q set to 1%. No values were classified as outliers.

Immunohistochemistry of Fixed Frozen Tissue Sections

Immediately after blood collection, mice were fixed by transcardial perfusion with 4% paraformaldehyde in 0.1M phosphate buffer pH 7.4. The brains were removed and post-fixed in the same fixative overnight, followed by cryoprotection in 30% sucrose and embedding in Tissue-Tek® O.C.T.™ compound (Sakura Finetek; Cat#4583). The brains were then coronally sectioned using a cryostat (Leica CM1950; RRID:SCR_018061) at 14 µm thickness for immunohistochemistry using standard techniques, as previously described [1,4], to assess the expression levels and localization of specific axonal and glial proteins. The interhemispheric midline crossing of the anterior commissure served as a reference marker of the injury epicenter (bregma 0) in the stained rostral coronal sections. Sections under the impact site (± 0.1 mm from bregma) were immunolabeled for β-APP to identify damaged axons within the CC. More caudal sections through the hippocampus (-1.5 ± 0.2 mm from bregma) were immunolabeled with glial fibrillary acidic protein (GFAP) to assess astrocyte reactivity in response to abnormal neuronal activity, along with ionized calcium binding associated protein 1 (IBA1) to detect reactive microglia. Cell nuclei were labeled with 4,6 diamino2-phenylindole (DAPI; Sigma-Aldrich, Cat#D9542). A list of primary and secondary antibodies is provided in Supplementary **Tables S2** and **S3**.

Analysis of Axon Damage in the CC

β-APP immunoreactivity is widely recognized as a marker for axonal damage [5-7], and we have previously reported that it labels damaged axons in this TBI model in mice [1,8,9]. Coronal β-APP-immunostained sections were photographed using a Zeiss LSM 700 confocal microscope with a 20x/0.8 oil Plan-Apochromat objective. The CC region contained within the entire z-stack of 640.17 μm × 416.99 μm × 6-10 μm acquired at 1 µm intervals was used for subsequent quantification. Z-stack depth varied from 6 to 10 µm to accommodate differences in β-APP distribution between sham and TBI mice. The number of β-APP immunolabeled axons per unit volume of CC was manually assessed from maximum intensity projection images of confocal z-stacks using the ImageJ software (RRID:SCR_003070). β-APP-positive axonal profiles, defined as axons with swollen regions showing increased diameter (either singly or in succession) and/or axons with terminal-bulb like structures, were counted as previously described [10]. Two images per animal (one image per brain hemisphere) were analyzed from equivalent regions of the medial CC of each hemisphere and averaged to yield a single measure per animal. Profiles with accumulated β-APP immunoreactivity were counted as damaged axons per imaged volume (mm^3^).

Analysis of Glial Reactivity in the Hippocampus

Reactive gliosis in the hippocampus, a region highly susceptible to seizure activity, was evaluated by immunostaining for GFAP in astrocytes and IBA1 in microglia/macrophages. Wide-field images of the hippocampal region were acquired with a 4x objective on an Olympus IX70 inverted fluorescence microscope (RRID:SCR_018604) using a SPOT R3 camera. Similar regions of the hippocampus were selected for imaging in each mouse.

Single-channel epifluorescence images were processed in ImageJ for conversion to grayscale, followed by tracing of the hippocampus region-of-interest (ROI) in one brain hemisphere per animal. Astrogliosis and microglia/macrophage activation were estimated from the percentage of GFAP and IBA1 immunoreactive areas within the outlined hippocampal region using the Threshold and Analyze Particles functions in ImageJ as described previously [8,11].

Supplementary One-Day Post-Injury Study

While the incidence of new acute symptomatic seizures is increased during the first week post-injury, the highest incidence is during the first 24 hours after TBI [12]. Therefore, seizure behavior at 24 hours after TBI was examined in additional 8-week-old C57BL/6J male and female cohorts (**Figure S2**). Mice received either sham or TBI procedures followed by a single i.p. injection of 4-AP (0.5 or 5 mg/kg) or saline vehicle (**Table S1**) approximately 24 hours after surgery. Experiments and assessments were conducted as described above in Study 2. As 4-AP treatment requires administration beyond day 3 post-injury to induce beneficial effects on axon damage by electron microscopy [1], the purpose of this one-day post-injury study focused solely on seizure assessment.

**Statistical Analysis for Studies 1 and 2**

Statistical analyses were conducted using GraphPad Prism v10.4.1 (RRID:SCR_002798), with a significance level of an p < 0.05. Nodal domain organization data were analyzed using one-way ANOVA with Holm-Sidak post hoc correction for multiple comparisons. Statistical analyses for experiments assessing seizure risk were performed using two-way ANOVA to evaluate the effects of injury and drug treatment dosage, or sex and experimental group as appropriate. Post-hoc Holm-Sidak multiple comparisons test was applied to identify group differences following significant main or interaction effects. Final figures were set up using Adobe Photoshop (RRID:SCR_014199).

**Study 3: Genome-Wide Spatial Transcriptomics**

4-AP Treatment Regimen

Male C57BL/6J mice received either vehicle (0 mg/kg) or low dose (0.5 mg/kg) 4-AP for 6 consecutive days (*b.i.d.*, i.p.) starting one day after surgical procedures and ending with a final injection on day 7 (**Table S1**). After the final morning injection of day 7 post-surgical procedures, mice were transcardially perfused with 10% formalin followed by post-fixation of the extracted brain in the same fixative at 4° C for a maximum of 3 days.

Spatial Transcriptomics and Sequencing:

Each sample included in the spatial transcriptomics analysis had a DV200 greater than 50%, measured using the Fragment Analyzer 5200 (Agilent; Cat#M5310AA) and DNF-472 High Sensitivity RNA Kit (Agilent, Cat#DNF-472-FR). Coronal FFPE brain tissue sections (7 µm) were prepared for Visium CytAssist spatial transcriptomics in accordance with 10x Genomics protocols CG000518 and CG000520. Digital brightfield images of hematoxylin and eosin (H&E) histological sections were acquired with a Zeiss Axioscan Z1 (RRID:SCR_020927). Then, cDNA barcoded libraries were generated using the Visium CytAssist Spatial Gene Expression Reagents and Kits for the mouse FFPE tissue (10x Genomics for Mouse Transcriptome, 11 mm, 4 rxns, PN-1000523), Visium CytAssist Reagent Accessory Kit (PN-1000499 with Handbook CG000495) and the Dual Index Kit TS Set A (PN-1000251). Indexed barcoded libraries were then pooled and sequenced using a high-throughput Illumina NovaSeq X Plus with NovaSeq X Series 10B Reagent Kits (Illumina, Cat#20085596). Tissue sections included in analysis met the following criteria: greater than 50,000 UMI count range, symmetrical UMIs, greater than 6,500 median genes per spot, greater than 50% sequencing saturation, greater than 890,000,000 reads per sample.

Spatial Transcriptomics Data Analysis

The transcriptome in each FFPE sample was mapped to the mouse genome mm10. All other parameters for generating the count data for spatial transcriptomes were set to their default settings. Quality assessment metrics were calculated and verified for the 24 spatial transcriptomes. Spots were removed due to extremely low UMI counts, low gene counts, high percentages of mitochondrial genes (outliers), and isolated away from the main tissue sections. Genes that were expressed in fewer than three spots, as well as mitochondrial and ribosomal genes, were filtered outWe identified 2,000 highly variable genes based on their expression means and variances. Using these genes, we conducted principal components analysis (PCA) to project the spots into a low-dimensional space defined by the first 20 principal components (PCs). After this, we used the corrected PC matrices to perform unsupervised shared-nearest-neighbor (SNN) clustering with a resolution of 0.5. We also conducted visualization analysis using either Uniform Manifold Approximation and Projection (UMAP) or t-Distributed Stochastic Neighbor Embedding (t-SNE). To identify sub-clusters within the region of interest, we passed marker genes into the Seurat RunPCA function. Subsequently, Seurat applied the Louvain algorithm on the SNN graph created in the PCA space to categorize the spots.

Differential expression analysis

Gene counts from the mouse brain datasets are normalized using barcode-specific factors. These factors are derived from the median ratio of gene counts relative to the geometric mean for each gene. Subsequently, we conducted differential expression analysis using the “DESeq” function, which employs a negative binomial distribution and Wald statistics for the analysis. Nominal P-values were adjusted for multiple testing using the Benjamini-Hochberg (BH) method. Genes were classified as differentially expressed (DE) if the fold change (FC) was ≥ 0.58 and the adjusted P-value was ≤ 0.1. For each gene analyzed, we report the following: baseMean (the mean of normalized counts), LFC, lfcSE (the standard error of the LFC estimate), P value (the Wald test P-value), and Padj (the BH-adjusted P-value). A notation of NA indicates missing values.

Spatial mapping of cell states with cell2location

To spatially map mouse brain cell types defined by single-cell transcriptomics, Visium data was mapped to tissue section spots using cell2location (v0.1.3) [13]. Briefly, we first estimated reference expression signatures for major cell types in healthy adult mouse brain using regularized negative binomial regressions and 2.3 million single-cell transcriptomes from The Allen Brain Cell Atlas [14]. The reference cell state signatures estimated by cell2location were then used for mapping cell types to corresponding regions, allowing for the estimation of the abundance of each cell state in each Visium spot by decomposing its mRNA counts. Each slide was deconvoluted using hierarchical Bayesian models, as implemented in the run_cell2location function. H&E images of the Visium slides were used to determine the average number of nuclei per Visium spot (n = 20) in the tissue, which was then used as a hyperparameter in the cell2location pipeline. We then calculated cell-type proportions using the cell-type-specific abundance estimations for each spot in the Visium sections with the inferred reference cell state signatures. The cell-type compositions for the entire slide were calculated by summing the estimated number of cells of each type across all spots. A spatial ScatterPie graphic was created to display the composition of each cell type as estimated by the cell2location deconvolution method, with each scatter point representing a spot in the Visium spatial transcriptomics data from the mouse brain.

Pathway Analysis and Gene set enrichment analysis (GSEA)

GSEA was conducted using the R package fGSEA (v1.34.2)[15] to identify enriched gene sets. The analysis includes both customized, published [16,17], and publicly available MSigDB (<https://www.gsea-msigdb.org/gsea/msigdb>) gene sets for cell phenotypes and molecular pathways. The fGSEA method utilizes a ranked list derived from DESeq2 outputs, with the ranking determined by the 'stat' column from the DESeq2 results.

Signature scores

Signatures were extracted from the cited literature and uploaded to AUCell [18]. Scores for each spot were calculated using AUCell method. For each input gene list, AUCell computes a score for each spot by ranking all genes based on their expression levels in the cell and identifies what proportion of the genes from the gene list fall within the top 5% of expressed genes (default cutoff). The score values for each spot were then utilized for downstream analysis.

Immunohistochemistry of Formalin-fixed Paraffin-embedded (FFPE) Tissue Sections

Coronal sections (7 μm) anterior to the spatial transcriptomics sections and under the TBI impact site were immunolabeled for myelin basic protein (MBP), beta-amyloid precursor protein (β−APP), glial fibrillary acidic protein (GFAP), or ionized calcium-binding adaptor molecule 1 (IBA1) (**Table** **S2**). Immunohistochemistry was conducted by FD-Neurotechnologies (Columbia, MD). The immunoreaction product was visualized according to the avidin-biotin complex method [19] using the Vectastain elite ABC kit (Vector Lab., Burlingame, CA) and 3’,3’-diaminobenzidine (Sigma, St. Louis, MO) as a chromogen.

**SUPPLEMENTARY TABLES and FIGURES**

**Supplementary Table S1. Summary of animal group allocation across studies.**

| **Strain** | **Total Number of Mice** | **Surgery** | **Treatment** | **Study Endpoint** | **Study** |
| --- | --- | --- | --- | --- | --- |
| Thy1-YFP-16^a^ | n = 8 | sham | 0 mg/mg 4-AP (n = 4; 2 males, 2 females)  0.5 mg/kg 4-AP (n = 4; 2 males, 2 females) | 7 days post-injury | Node of Ranvier Organization Study |
|  | n = 8 | TBI | 0 mg/mg 4-AP (n = 4; 2 males, 2 females)  0.5 mg/kg 4-AP (n = 4; 2 males, 2 females) |  |  |
| C57BL/6J | n = 24 | sham | 0 mg/mg 4-AP (n = 8; 4 males, 4 females)  0.5 mg/kg 4-AP (n = 8; 4 males, 4 females)  5 mg/kg 4-AP (n = 8; 4 males, 4 females) | 7 days post-injury | Seizure Risk and 4-AP Dosing Study |
|  | n =24 | TBI | 0 mg/mg 4-AP (n = 8; 4 males, 4 females)  0.5 mg/kg 4-AP (n = 8; 4 males, 4 females)  5 mg/kg 4-AP (n = 8; 4 males, 4 females) |  |  |
| C57BL/6J | n = 24 | sham | 0 mg/mg 4-AP (n = 8; 4 males, 4 females)  0.5 mg/kg 4-AP (n = 8; 4 males, 4 females)  5 mg/kg 4-AP (n = 8; 4 males, 4 females) | 1 day post-injury | Supplementary Seizure Risk Study |
|  | n = 24 | TBI | 0 mg/mg 4-AP (n = 8; 4 males, 4 females)  0.5 mg/kg 4-AP (n = 8; 4 males, 4 females)  5 mg/kg 4-AP (n = 8; 4 males, 4 females) |  |  |
| C57BL/6J | n = 12 | sham | 0 mg/mg 4-AP (n = 6 males)  0.5 mg/kg 4-AP (n = 6 males) | 7 days post-injury | Genome-Wide Spatial Transcriptomics Study |
|  | n = 12 | TBI | 0 mg/mg 4-AP (n = 6 males)  0.5 mg/kg 4-AP (n = 6 males) |  |  |

*n* indicates the number of independent biological replicates (animals) included in the statistical analyses described in Methods.

^a^Mice previously reported in Radomski et al., 2022. The current study reports novel quantitative analyses of nodal domains in confocal microscopy images previously obtained but not included in the prior publication.

**Supplementary Table S2. List of primary antibodies used for immunohistochemistry.**

| **Antibody** | **Company, Catalog Number, RRIDs** | **Dilution** |
| --- | --- | --- |
| Rabbit polyclonal anti-Nav1.6 | Alomone Labs  Cat # ASC-009, RRID:AB_2040202 | 1:100 |
| Mouse monoclonal anti-Caspr | UC Davis/NIH NeuroMab Facility  Cat # K65/35, RRID:AB_2877274 | 1:500 |
| Mouse monoclonal anti-Kv1.2 | UC Davis/NIH NeuroMab Facility  Cat # K14/K16, RRID:AB_2877295 | 1:300 |
| Rabbit polyclonal anti-Caspr | Abcam  Cat # ab34151, RRID:AB_869934 | 1:500  1:1,500^a^ |
| Rabbit polyclonal anti-beta Amyloid (CT695)^a^ | Thermo Fisher Scientific  Cat# 51-2700, RRID:AB_2533902 | 1:100  1:1,200^a^ |
| Chicken polyclonal anti-GFAP | Abcam  Cat # ab4674, RRID:AB_304558 | 1:1,000 |
| Rabbit polyclonal anti-IBA1^a^ | FUJIFILM Wako Pure Chemical Corporation  Cat # 019-19741, RRID:AB_839504 | 1:500  1:1,200^a^ |
| Rat monoclonal anti-GFAP^a^ | Thermo Fisher Scientific  Cat# 13-0300, RRID:AB_2532994 | 1:1,200^a^ |
| Rat monoclonal anti-MBP^a^ | Millipore  Cat # MAB386, RRID:AB_94975 | 1:200^a^ |
| Rabbit polyclonal anti-Olig2^a^ | Millipore  Cat# AB9610, RRID:AB_570666 | 1:400^a^ |
| Rabbit polyclonal anti-BCAS1^a^ | Synaptic Systems  Cat # 445 003, RRID:AB_2864793 | 1:1,200^a^ |

RRID: Research Resource Identifiers, rrids.org. Antigen retrieval procedures for the following double IHC experiments: 0.3M glycine for 30 min at room temperature for sections stained with rabbit anti-Nav1.6 and mouse anti-Caspr; Tris-EDTA buffer (pH 9) for 15 min at 90 °C for sectioned stained with mouse anti-Kv1.2 and rabbit anti-Caspr; 10 mM EDTA (pH 6) for 10 min at 95 °C for sections stained with chicken anti-GFAP and rabbit anti-IBA1. ^a^Antibodies and dilutions used by FD Neurotechnologies for FFPE tissue sections with spatial transcriptomics mice.

**Supplementary Table S3. List of secondary antibodies used for immunohistochemistry performed in-house.**

| **Antibody** | **Company, Catalog Number, RRIDs** | **Dilution** |
| --- | --- | --- |
| Donkey anti-rabbit Alexa Fluor 647 | Jackson ImmunoResearch Labs  Cat # 711-606-152, RRID:AB_2340625 | 1:300 |
| Donkey anti-mouse Alexa Fluor 594 | Jackson ImmunoResearch Labs  Cat # 715-586-151, RRID:AB_2340858 | 1:300 |
| Donkey anti-rabbit Cy3 | Jackson ImmunoResearch Labs  Cat # 711-166-152, RRID:AB_ 2313568 | 1:100 |
| Donkey anti-chicken Alexa Fluor 488 | Jackson ImmunoResearch Labs  Cat # 703-545-155, RRID:AB_ 2340375 | 1:400 |
| Donkey anti-rabbit Alexa Fluor 594 | Jackson ImmunoResearch Labs  Cat # 711-587-003, RRID:AB_2340623 | 1:400 |

RRID: Research Resource Identifiers, rrids.org.

**Supplementary Table S4. Summary of statistical analyses associated with Figure 1.**

|  |  | One-way ANOVA (α < 0.05) | | | Holm-Sidak’s Multiple Comparisons Test (Adjusted p value) | | |
| --- | --- | --- | --- | --- | --- | --- | --- |
| **Fig.** | **Parameter** | | **Test Statistic** | **P value** | **Sham Veh vs TBI Veh** | **Sham 4-AP vs TBI 4-AP** | **TBI Veh vs TBI 4-AP** |
| 1A | Nav1.6 Nodal Area | | F (3,12) = 4.066 | p = 0.0330 | p = 0.0426 | p = 0.9837 | p = 0.0426 |
| 1A | Void Nodes | | F (3,12) = 10.50 | p = 0.0011 | p = 0.0017 | p = 0.0250 | p = 0.1147 |
| 1A | Heminodes | | F (3,12) = 32.64 | p < 0.0001 | p < 0.0001 | p = 0.0242 | p = 0.0001 |
| 1B | Mislocalized Kv1.2 | | F (3,12) = 8.851 | p = 0.0023 | p = 0.0027 | p = 0.0418 | p = 0.2075 |

Abbreviations: Veh = vehicle saline; 4-AP = 0.5 mg/kg 4-aminopyridine.

**Supplementary Table S5. Summary of statistical analyses associated with Figure 2.**

| **Figure 2B: β-APP^+^ Damaged Axons in CC** | **Figure 2C: Serum NfL Injury Biomarker** |
| --- | --- |
| **Test Statistic (Main Factors: Injury vs Drug Dosage)**  F injury^a^ x drug dosage^b^ (2,41) = 5.334, p = 0.0087  F injury (1, 41) = 142.4, p < 0.0001  F drug dosage (2, 41) = 5.678, p = 0.0067 | **Test Statistic (Main Factors: Injury vs Drug Dosage)**  F injury x drug dosage (2,42) = 0.1033, p = 0.9021  F injury (1, 42) = 33.75, p < 0.0001  F drug dosage (2, 42) = 0.4978, p = 0.6114 |
| *Holm-Sidak’s Multiple Comparisons Test (Adjusted p value):* | *Holm-Sidak’s Multiple Comparisons Test (Adjusted p value):* |
| Sham 0 mg/kg 4-AP vs TBI 0 mg/kg 4-AP: p < 0.0001  Sham 0.5 mg/kg 4-AP vs TBI 0.5 mg/kg 4-AP: p < 0.0001  Sham 5 mg/kg 4-AP vs TBI 5 mg/kg 4-AP: p < 0.0001 | Sham 0 mg/kg 4-AP vs TBI 0 mg/kg 4-AP: p = 0.0054  Sham 0.5 mg/kg 4-AP vs TBI 0.5 mg/kg 4-AP: p = 0.0054  Sham 5 mg/kg 4-AP vs TBI 5 mg/kg 4-AP: p = 0.0017 |
| Sham 0 mg/kg 4-AP vs Sham 0.5 mg/kg 4-AP: p > 0.9999  Sham 0 mg/kg 4-AP vs Sham 5 mg/kg 4-AP: p > 0.9999  Sham 0.5 mg/kg 4-AP vs Sham 5 mg/kg 4-AP: p > 0.9999 | Sham 0 mg/kg 4-AP vs Sham 0.5 mg/kg 4-AP: p = 0.9835  Sham 0 mg/kg 4-AP vs Sham 5 mg/kg 4-AP: p = 0.9835  Sham 0.5 mg/kg 4-AP vs Sham 5 mg/kg 4-AP: p = 0.9835 |
| TBI 0 mg/kg 4-AP vs TBI 0.5 mg/kg 4-AP: p = 0.0008  TBI 0 mg/kg 4-AP vs TBI 5 mg/kg 4-AP p = 0.0009  TBI 0.5 mg/kg 4-AP vs TBI 5 mg/kg 4-AP: p > 0.9999 | TBI 0 mg/kg 4-AP vs TBI 0.5 mg/kg 4-AP: p = 0.9835  TBI 0 mg/kg 4-AP vs TBI 5 mg/kg 4-AP p = 0.9835  TBI 0.5 mg/kg 4-AP vs TBI 5 mg/kg 4-AP: p = 0.9835 |
| **Test Statistic (Main Factors: Sex vs Experimental Group)**  F sex^c^ x drug experimental group^d^ (5,35) = 0.3089, p = 0.9043  F sex (1, 35) = 0.001188, p = 0.9727  F experimental group (5, 35) = 5.678, p < 0.0001 | **Test Statistic (Main Factors: Sex vs Experimental Group)**  F sex x drug experimental group (5,36) = 0.1475, p = 0.9795  F sex (1, 36) = 0.6384, p = 0.4295  F experimental group (5, 36) = 6.220, p = 0.0003 |
| *Holm-Sidak’s Multiple Comparisons Test (Adjusted p value):* | *Holm-Sidak’s Multiple Comparisons Test (Adjusted p value):* |
| Sham 0 mg/kg 4-AP: Males vs Females, p = 0.9944  Sham 0.5 mg/kg 4-AP: Males vs Females, p = 0.9944  Sham 5 mg/kg 4-AP: Males vs Females, p = 0.9944 | Sham 0 mg/kg 4-AP: Males vs Females, p = 0.9985  Sham 0.5 mg/kg 4-AP: Males vs Females, p = 0.9825  Sham 5 mg/kg 4-AP: Males vs Females, p = 0.9885 |
| TBI 0 mg/kg 4-AP: Males vs Females, p = 0.9878  TBI 0.5 mg/kg 4-AP: Males vs Females, p = 0.9944  TBI 5 mg/kg 4-AP: Males vs Females, p = 0.9194 | TBI 0 mg/kg 4-AP: Males vs Females, p = 0.9390  TBI 0.5 mg/kg 4-AP: Males vs Females, p = 0.9885  TBI 5 mg/kg 4-AP: Males vs Females, p = 0.9885 |
| **Figure 2D: Racine Seizure Behavior Score** | **Figure 2F: GFAP^+^ Area in Hippocampus** |
| **Test Statistic (Main Factors: Injury vs Drug Dosage)**  F injury x drug dosage (2,42) = 0.1489, p = 0.8621  F injury (1, 42) = 0.1489, p = 0.7015  F drug dosage (2, 42) = 1180, p < 0.0001 | **Test Statistic (Main Factors: Injury vs Drug Dosage)**  F injury x drug dosage (2,18) = 1.120, p = 0.3481  F injury (1, 18) = 5.914, p = 0.0257  F drug dosage (2, 18) = 26.28, p < 0.0001 |
| *Holm-Sidak’s Multiple Comparisons Test (Adjusted p value):* | *Holm-Sidak’s Multiple Comparisons Test (Adjusted p value):* |
| Sham 0 mg/kg 4-AP vs TBI 0 mg/kg 4-AP: p > 0.9999  Sham 0.5 mg/kg 4-AP vs TBI 0.5 mg/kg 4-AP: p > 0.9999  Sham 5 mg/kg 4-AP vs TBI 5 mg/kg 4-AP: p = 0.8805 | Sham 0 mg/kg 4-AP vs TBI 0 mg/kg 4-AP: p = 0.0643  Sham 0.5 mg/kg 4-AP vs TBI 0.5 mg/kg 4-AP: p = 0.3736  Sham 5 mg/kg 4-AP vs TBI 5 mg/kg 4-AP: p = 0.6943 |
| Sham 0 mg/kg 4-AP vs Sham 0.5 mg/kg 4-AP: p > 0.9999  Sham 0 mg/kg 4-AP vs Sham 5 mg/kg 4-AP: p < 0.0001  Sham 0.5 mg/kg 4-AP vs Sham 5 mg/kg 4-AP: p < 0.0001 | Sham 0 mg/kg 4-AP vs Sham 0.5 mg/kg 4-AP: p = 0.7350  Sham 0 mg/kg 4-AP vs Sham 5 mg/kg 4-AP: p = 0.0002  Sham 0.5 mg/kg 4-AP vs Sham 5 mg/kg 4-AP: p = 0.0005 |
| TBI 0 mg/kg 4-AP vs TBI 0.5 mg/kg 4-AP: p > 0.9999  TBI 0 mg/kg 4-AP vs TBI 5 mg/kg 4-AP p < 0.0001  TBI 0.5 mg/kg 4-AP vs TBI 5 mg/kg 4-AP: p < 0.0001 | TBI 0 mg/kg 4-AP vs TBI 0.5 mg/kg 4-AP: p = 0.7350  TBI 0 mg/kg 4-AP vs TBI 5 mg/kg 4-AP p = 0.0112  TBI 0.5 mg/kg 4-AP vs TBI 5 mg/kg 4-AP: p = 0.0031 |
| **Test Statistic (Main Factors: Sex vs Experimental Group)**  F sex x drug experimental group (5,36) = 0.1304, p = 0.9844  F sex (1, 36) = 0.1304, p = 0.7201  F experimental group (5, 36) = 413.3, p < 0.0001 | **Test Statistic (Main Factors: Sex vs Experimental Group)**  Not analyzed^e^. |
| *Holm-Sidak’s Multiple Comparisons Test (Adjusted p value):* |  |
| Sham 0 mg/kg 4-AP: Males vs Females, p > 0.9999  Sham 0.5 mg/kg 4-AP: Males vs Females, p > 0.9999  Sham 5 mg/kg 4-AP: Males vs Females, p > 0.9999 |  |
| TBI 0 mg/kg 4-AP: Males vs Females, p > 0.9999  TBI 0.5 mg/kg 4-AP: Males vs Females, p > 0.9999  TBI 5 mg/kg 4-AP: Males vs Females, p = 0.9444 |  |

Statistical analysis performed with two-way ANOVA followed by Holm-Sidak’s multiple comparisons test.

^a^Injury = sham or TBI; ^b^Drug Dosage = 0, 0.5 or 5 mg/kg 4-aminopyridine (4-AP). ^c^Sex = male or female; ^d^Experimental Group = Sham + 0 mg/kg 4-AP; Sham + 0.5 mg/kg 4-AP; Sham + 5 mg/kg 4-AP; TBI + 0 mg/kg 4-AP; TBI + 0.5 mg/kg 4-AP; TBI + 5 mg/kg 4-AP. ^e^Not Analyzed: sex not included as a factor in the analysis of the GFAP data that had a n of four mice (2 males, 2 females) per group. Abbreviations: β-APP: β-amyloid precursor protein; CC: corpus callosum; NfL: neurofilament light chain; GFAP: glial fibrillary associated protein.

**Supplementary Figure S1.**


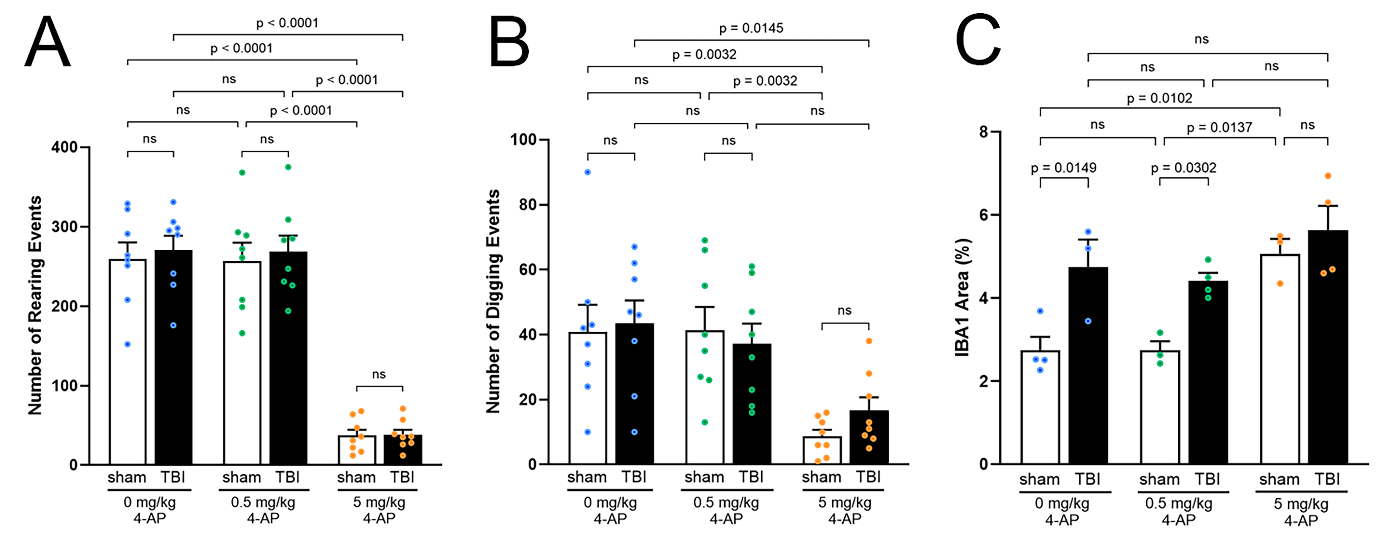


**Figure S1. *Effects of 4-AP treatment post-TBI on digging and rearing behaviors and hippocampal microglial activation at 7 days post-injury*.** Number of exploratory rearing (**A**) and digging (**B**) events during a 1-hour video recording session in sham and TBI mice treated with either 0, 0.5 or 5 mg/kg 4-AP (n = 8 per group; 4 males, 4 females). Concomitant with seizure behavior activity, mice receiving the 10x higher dose of 4-AP (5 mg/kg) showed significant reductions in rearing and digging behaviors. The low dose (0.5 mg/kg) 4-AP treatment did not interfere with these exploratory behaviors. (**C**) Microgliosis examined with IBA1 immunolabeling in the hippocampus (n = 3-4 mice per group; 1-2 males, 2 females) was increased in TBI versus sham in vehicle and 0.5 mg/kg treatment groups. Both sham and TBI mice treated with the higher 5 mg/kg 4-AP dose had significantly increased IBA1 immunoreactivity in the hippocampal region. Data points represent individual animals (mean ± SEM). Statistical analysis results are provided in Supplementary **Table S6**.

**Supplementary Table S6. Summary of statistical analyses associated with Supplementary Figure S1.**

| *7-day Time Point Data Analysis: Two-way ANOVA (α < 0.05)* | | | |
| --- | --- | --- | --- |
|  | **Suppl. Fig. 1A**  **Rearing Events** | **Suppl. Fig. 1B**  **Digging Events** | **Suppl. Fig. 1C**  **IBA1 IHC** |
| F (injury^a^ x drug dosage^b^) | F (2, 42) = 0.0630 p = 0.9390 | F (2, 42) = 0.4954 p = 0.6128 | F (2, 15) = 1.518 p = 0.2510 |
| F (injury) | F (1, 42) = 0.3176 p = 0.5761 | F (1, 42) = 0.1780 p = 0.6752 | F (1, 15) = 16.151 p = 0.0011 |
| F (drug dosage) | F (2, 42) = 115.4 p < 0.0001 | F (2, 42) = 13.94 p < 0.0001 | F (2, 15) = 10.36 p = 0.0015 |
| *Holm-Sidak’s Multiple Comparisons Test (Adjusted p value)* | | | |
| Sham Vehicle x TBI Vehicle | p = 0.95 | p = 0.8621 | p = 0.0149 |
| Sham Low-Dose 4-AP x TBI Low-Dose 4-AP | p = 0.95 | p = 0.8621 | p = 0.0302 |
| Sham High-Dose 4-AP x TBI High Dose 4-AP | p = 0.9715 | p = 0.7432 | p = 0.3654 |
| Sham Vehicle x Sham Low-Dose 4-AP | p = 0.994 | p = 0.9546 | p = 0.9922 |
| Sham Vehicle x Sham High-Dose 4-AP | p < 0.0001 | p = 0.0032 | p = 0.0102 |
| Sham Low-Dose 4-AP x Sham High-dose 4-AP | p < 0.0001 | p = 0.0032 | p = 0.0137 |
| TBI Vehicle x TBI Low-Dose 4-AP | p = 0.994 | p = 0.718 | p = 0.8278 |
| TBI Vehicle x TBI High-Dose 4-AP | p < 0.0001 | p = 0.0145 | p = 0.4191 |
| TBI Low-Dose 4-AP x TBI High-dose 4-AP | p < 0.0001 | p = 0.069 | p = 0.1712 |

Statistical analysis performed with two-way ANOVA followed by Hold-Sidak’s multiple comparisons test.

^a^Injury = sham or TBI; ^b^Drug Dosage = 0 (vehicle). 0.5 (low-dose), or 5 mg/kg (high-dose) 4-AP.

**Supplementary Figure S2.**


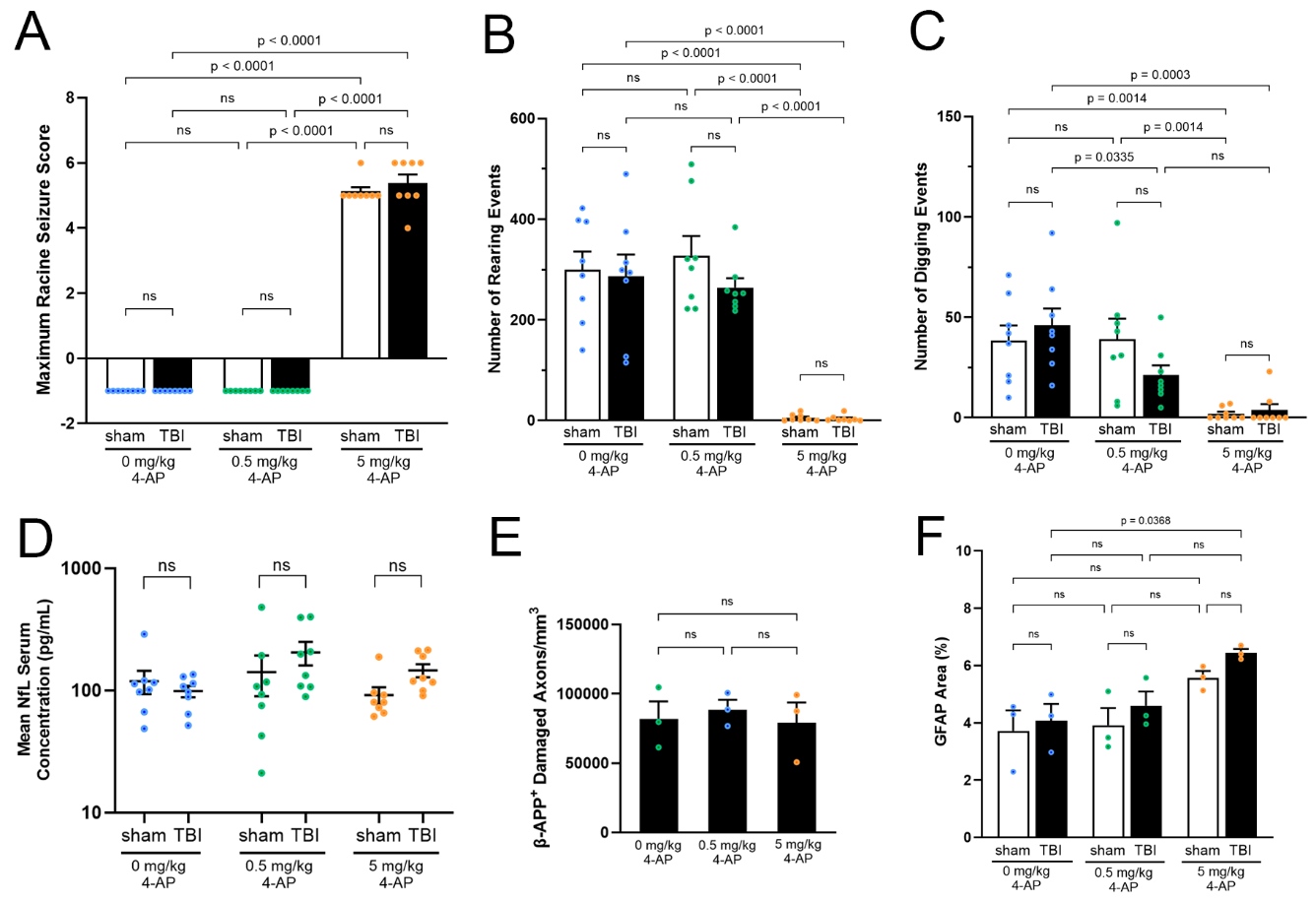


**Figure S2. *Supplemental seizure study at 1 day post-injury.*** (**A**) Maximum Racine seizure scores during 1-hour video monitoring following a single dose of 4-AP. Only sham and TBI mice administered 5 mg/kg 4-AP displayed seizures, with scores significantly higher than all other groups. (**B-C**) Exploratory novel cage behaviors (rearing and digging) were reduced in both sham and TBI mice treated with the higher dose of 4-AP (5 mg/kg). (**D**) Serum Nf-L concentrations did not differ among the groups on day 1 post-injury (**E**) β-APP-positive damaged axons in the corpus callosum under the impact site were comparable across TBI groups, indicating similar levels of acute injury prior to treatment effects observed at later time points. (**F**) Astrogliosis examined with GFAP immunolabeling in the seizure-prone hippocampus region was significantly increased in TBI mice treated with 5 mg/kg 4-AP compared to vehicle-treated injured animals, even at this early time point and after receiving only a single high dose of 4-AP. (**A-D**) n = 8 mice per group (4 males, 4 females). (**E**) n = 3 mice per group (1 male, 2 females). (**F**) n = 3-4 mice per group (1-2 males, 2 females). Data points represent individual animals (mean ± SEM). Statistical analysis results are provided in Supplementary **Table S7**.

**Supplementary Table S7. Summary of statistical analyses associated with Supplementary Figure S2.**

| *1-day Time Point Data Analysis: Two-way ANOVA (α < 0.05)* | | | | | |
| --- | --- | --- | --- | --- | --- |
|  | **Suppl. Fig. 2A**  **Racine Score** | **Suppl. Fig. 2B**  **Rearing Events** | **Suppl. Fig. 2C**  **Digging Events** | **Suppl. Fig. 2D**  **Serum Nf-L** | **Suppl. Fig. 2F**  **GFAP IHC** |
| F (injury^a^ x drug  dosage^b^) | F (2, 42) = 0.7368 p = 0.4847 | F (2, 42) = 0.6447 p = 0.5299 | F (2, 42) = 0.3210 p = 0.7272 | F (2, 42) = 1.065 p = 0.3538 | F (2, 12) = 0.1354 p = 0.8747 |
| F (injury) | F (1, 42) = 0.7368 p = 0.3955 | F (1, 42) = 1.208 p = 0.2780 | F (1, 42) = 0.7081 p = 0.4048 | F (1, 42) = 1.583 p = 0.2153 | F (1, 12) = 2.348 p = 0.1514 |
| F (drug dosage) | F (2, 42) = 1842 p < 0.0001 | F (2, 42) = 66.27 p < 0.0001 | F (2, 42) = 18.67 p < 0.0001 | F (2, 42) = 2.395 p = 0.1035 | F (2, 12) = 9.919 p = 0.0029 |
| *Holm-Sidak’s Multiple Comparisons Test (Adjusted p value)* | | | | | |
| Sham Vehicle x TBI Vehicle | p > 0.9999 | p = 0.9392 | p = 0.665 | p = 0.6484 | p = 0.632 |
| Sham Low-Dose 4-AP x TBI Low-Dose 4-AP | p > 0.9999 | p = 0.3396 | p = 0.1747 | p = 0.4108 | p = 0.5963 |
| Sham High-Dose 4-AP x TBI High Dose 4-AP | p = 0.3740 | p = 0.9687 | p = 0.8323 | p = 0.4108 | p = 0.5709 |
| Sham Vehicle x Sham Low-Dose 4-AP | p > 0.9999 | p = 0.7457 | p = 0.9367 | p = 0.7233 | p = 0.783 |
| Sham Vehicle x Sham High-Dose 4-AP | p < 0.0001 | p < 0.0001 | p = 0.0014 | p = 0.7935 | p = 0.1146 |
| Sham Low-Dose 4-AP x Sham High-dose 4-AP | p < 0.0001 | p < 0.0001 | p = 0.0014 | p = 0.7935 | p = 0.116 |
| TBI Vehicle x TBI Low-Dose 4-AP | p > 0.9999 | p = 0.7457 | p = 0.0335 | p = 0.6613 | p = 0.7274 |
| TBI Vehicle x TBI High-Dose 4-AP | p < 0.0001 | p < 0.0001 | p = 0.0003 | p = 0.1242 | p = 0.0368 |
| TBI Low-Dose 4-AP x TBI High-dose 4-AP | p < 0.0001 | p < 0.0001 | p = 0.141 | p = 0.7233 | p = 0.1146 |
| *1-day Time Point (TBI only) Data Analysis: One-way ANOVA (α < 0.05)* | | | Holm-Sidak’s Multiple Comparisons Test (Adjusted p value) | | |
|  | **Parameter** | **Statistics** | **TBI Veh vs TBI Low Dose** | **TBI Low Dose x TBI High Dose** | **TBI Veh vs TBI High Dose** |
| Suppl. Figure 2E | β-APP^+^ Axons | F (2, 60) = 0.1751  p = 0.8435 | p = 0.929 | p = 0.929 | p = 0.929 |

Statistical analysis performed with either one-way or two-way ANOVA followed by Hold-Sidak’s multiple comparisons test.

^a^Injury = sham or TBI; ^b^Drug Dosage = 0 (vehicle). 0.5 (low-dose), or 5 mg/kg (high-dose) 4-AP.

**Supplementary Figure S3**

**
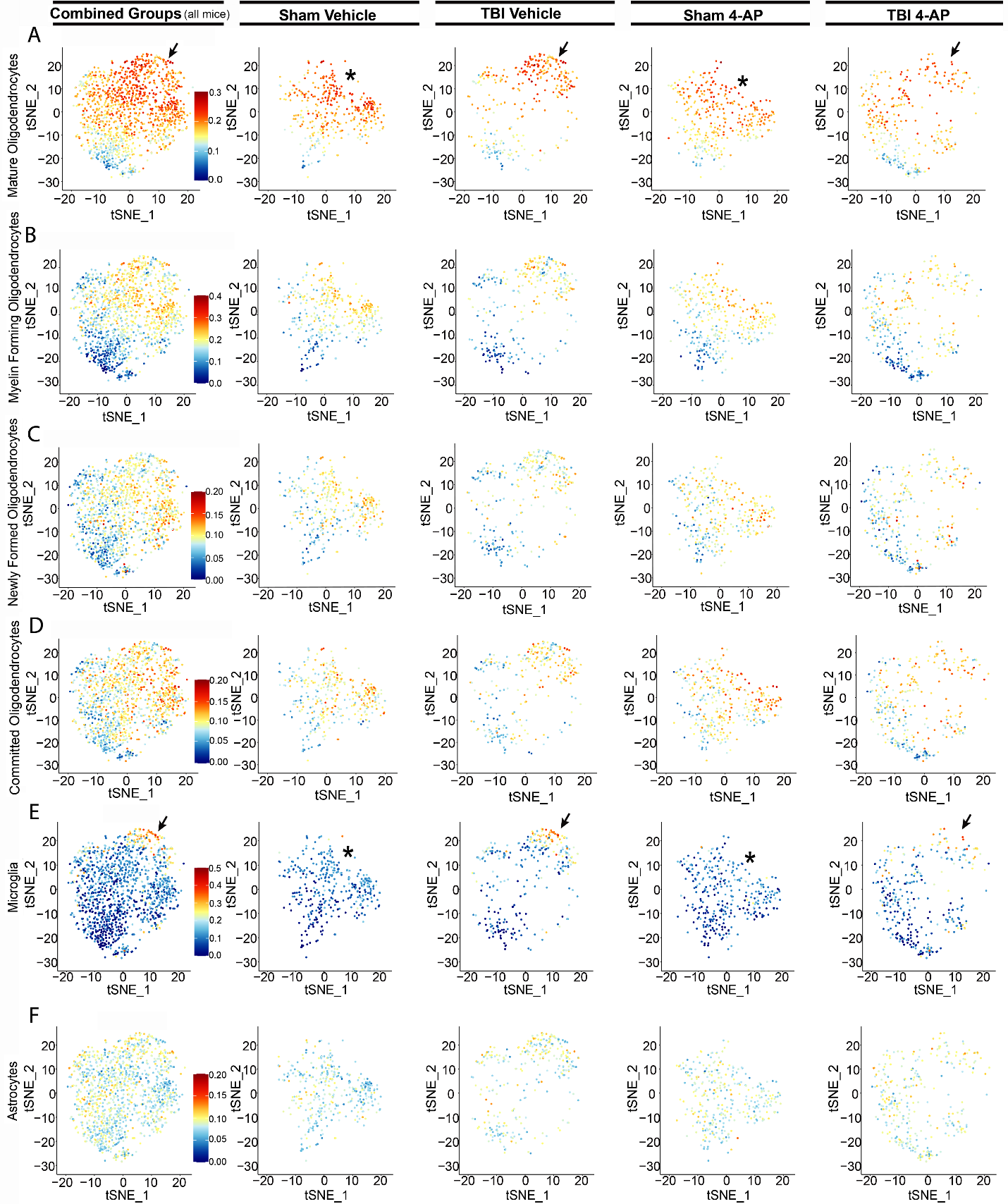
**

**Figure S3. *Glial gene set expression patterns within the corpus callosum clusters following injury and/or drug treatment****.*

Glial cell type gene set expression within corpus callosum (CC) spots of the five distinct clusters identified in **Figure 5** for genes shown in **Table S8**. The left column shows the distribution of CC spots combined from all 24 male mice (n = 6 per condition: sham or TBI with vehicle and sham or TBI with 0.5 mg/kg 4-AP).

**A-D:** Mature oligodendrocyte and earlier differentiation stages are broadly distributed within the CC spots, with mature oligodendrocyte gene sets most highly expressed. Following TBI, mature oligodendrocytes take on expression of disease-associated glial genes (arrows), that are minimal in non-injured mice (asterisks).

**E:** Microglia gene expression is highest in the disease-associated glia clusters of TBI mice (arrows).

**F:** Astrocyte gene expression is also broadly distributed within the CC spots.

**Supplementary Figure S4**

**
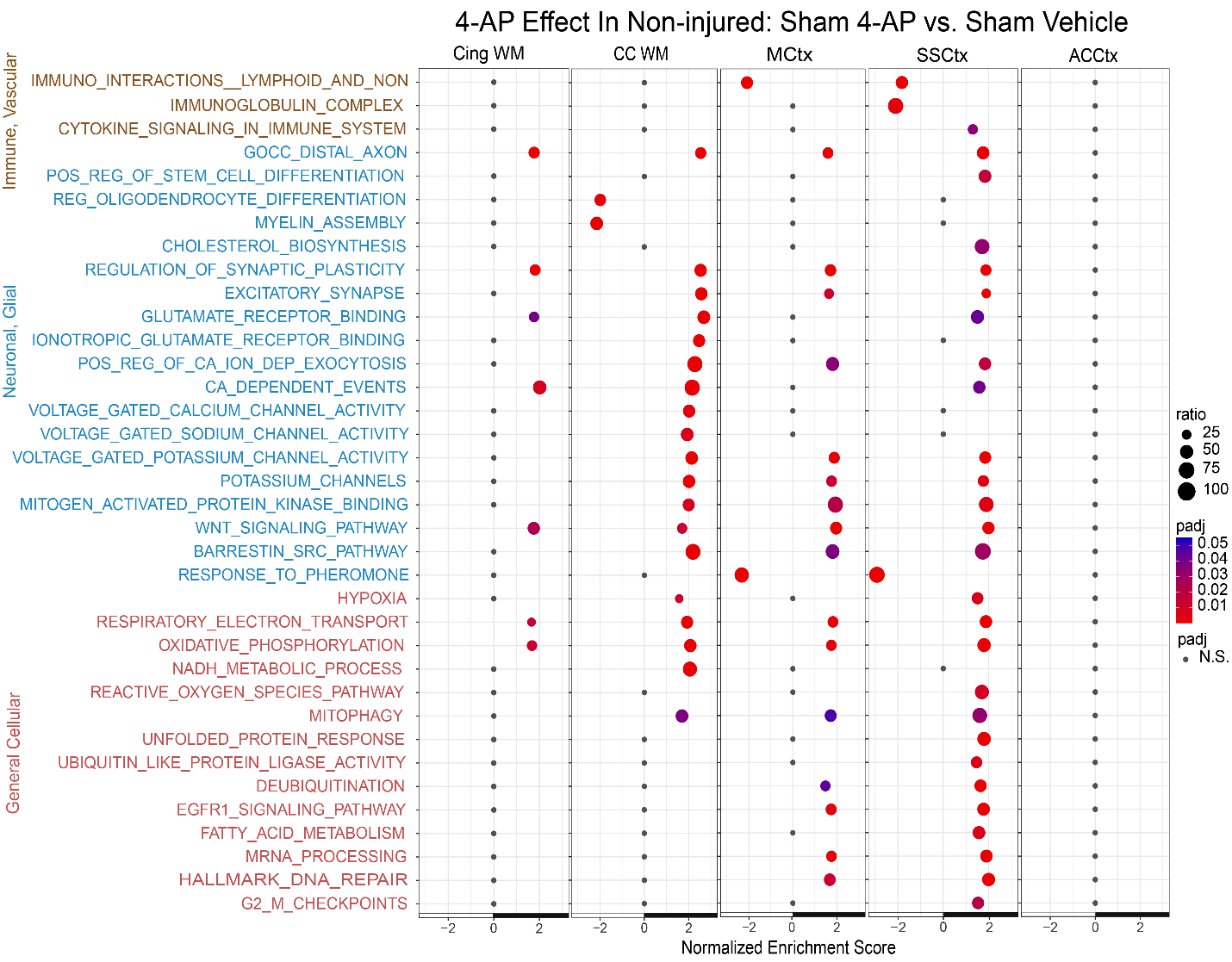
**

**Figure S4. *Gene set enrichment analysis (GSEA) identifies tissue response to 4-AP treatment, as compared to vehicle injections, in sham mice*.** Pairwise comparison of molecular pathway normalized enrichments scores between sham (non-injured) mice receiving either 4-AP (0.5 mg/kg) or saline vehicle injections with tissue collection on day 7. Pseudo-bulk ROI profiles compared for sham+4-AP (n = 6) relative to sham+vehicle (n = 6) male mice. See Supplementary Materials for the full set of significant pathways and leading-edge genes (**Table S11**).

**Supplementary Figure S5.**


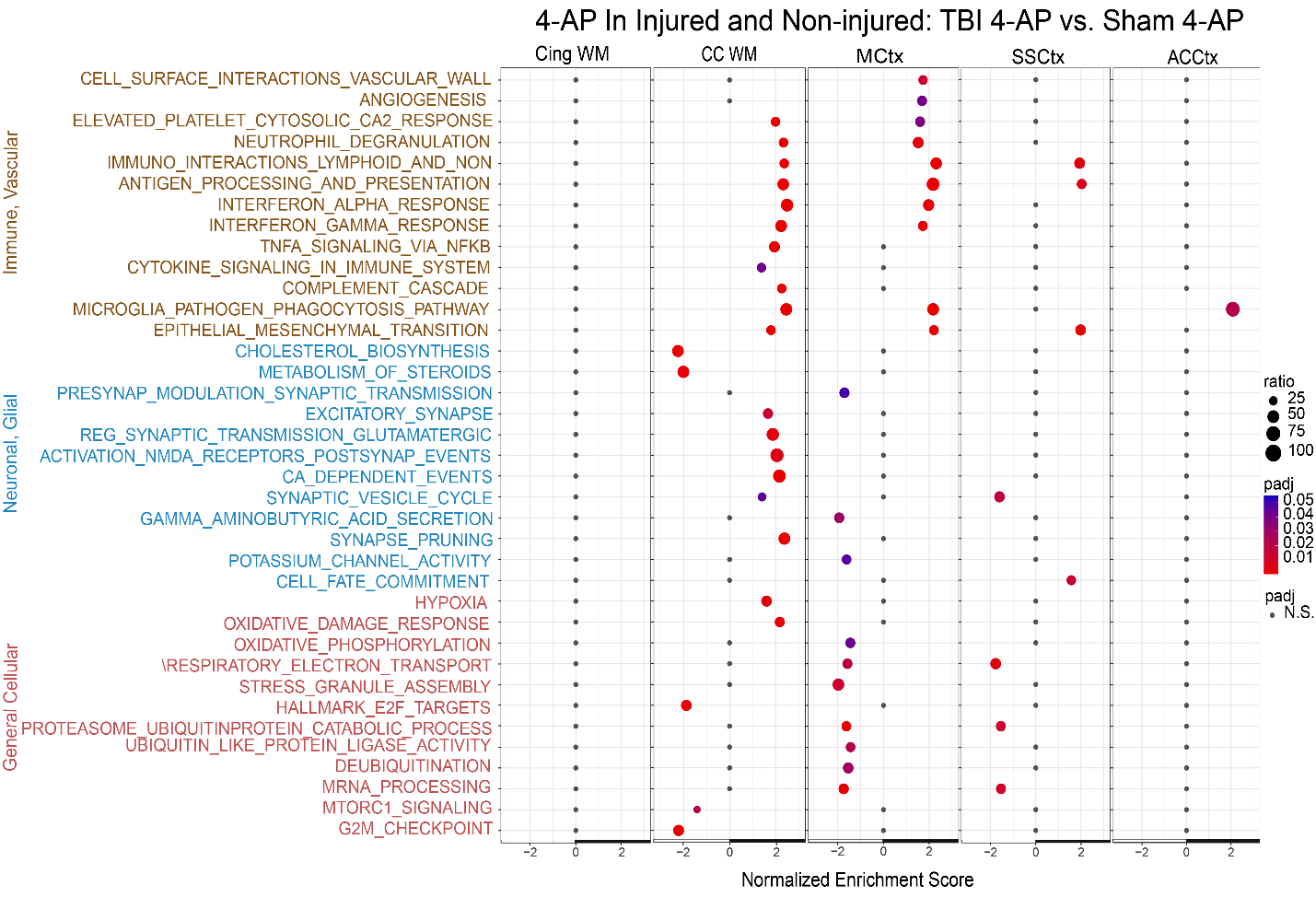


**Figure Supplemental S5. *Gene set enrichment analysis (GSEA) identifies tissue response to 4-AP treatment in TBI mice as compared to sham mice*.** Pairwise comparison of molecular pathway normalized enrichment scores between mice receiving 4-AP (0.5 mg/kg) after either TBI or sham procedures with tissue collection on day 7. Pseudo-bulk ROI profiles compared for TBI+4-AP (n = 6) relative to sham+4-AP (n = 6) male mice. See Supplementary Materials for details of the full set of significant pathways and leading-edge genes (**Table S11**).

**Supplementary References Cited**

1. Radomski, K.L. *et al.* (2022) Acute axon damage and demyelination are mitigated by 4-aminopyridine (4-AP) therapy after experimental traumatic brain injury. *Acta Neuropathol Commun* 10, 67. 10.1186/s40478-022-01366-z

2. Song, H. *et al.* (2022) Concussion leads to widespread axonal sodium channel loss and disruption of the node of Ranvier. *Acta Neuropathol* 144, 967-985. 10.1007/s00401-022-02498-1

3. Van Erum, J. *et al.* (2019) PTZ-induced seizures in mice require a revised Racine scale. *Epilepsy Behav* 95, 51-55. 10.1016/j.yebeh.2019.02.029

4. Marion, C.M. *et al.* (2018) Experimental Traumatic Brain Injury Identifies Distinct Early and Late Phase Axonal Conduction Deficits of White Matter Pathophysiology, and Reveals Intervening Recovery. *J Neurosci* 38, 8723-8736. 10.1523/JNEUROSCI.0819-18.2018

5. Stone, J.R. *et al.* (1999) The visualization of a new class of traumatically injured axons through the use of a modified method of microwave antigen retrieval. *Acta Neuropathol* 97, 335-345. 10.1007/s004010050996

6. Weber, M.T. *et al.* (2019) CLARITY reveals a more protracted temporal course of axon swelling and disconnection than previously described following traumatic brain injury. *Brain Pathol* 29, 437-450. 10.1111/bpa.12677

7. Xiong, G. *et al.* (2023) Detection and verification of neurodegeneration after traumatic brain injury in the mouse: Immunohistochemical staining for amyloid precursor protein. *Brain Pathol* 33, e13163. 10.1111/bpa.13163

8. Sullivan, G.M. *et al.* (2013) Oligodendrocyte lineage and subventricular zone response to traumatic axonal injury in the corpus callosum. *J Neuropathol Exp Neurol* 72, 1106-1125. 10.1097/NEN.0000000000000009

9. Yu, F. *et al.* (2017) Repetitive model of mild traumatic brain injury produces cortical abnormalities detectable by magnetic resonance diffusion imaging (DTI/DKI), histopathology, and behavior. *J Neurotrauma* 34, 1364-1381. 10.1089/neu.2016.4569

10. Song, S.K. *et al.* (2005) Demyelination increases radial diffusivity in corpus callosum of mouse brain. *Neuroimage* 26, 132-140. 10.1016/j.neuroimage.2005.01.028

11. Mierzwa, A.J. *et al.* (2015) Components of myelin damage and repair in the progression of white matter pathology after mild traumatic brain injury. *J Neuropathol Exp Neurol* 74, 218-232. 10.1097/NEN.0000000000000165

12. Ritter, A.C. *et al.* (2016) Incidence and risk factors of posttraumatic seizures following traumatic brain injury: A Traumatic Brain Injury Model Systems Study. *Epilepsia* 57, 1968-1977. 10.1111/epi.13582

13. Kleshchevnikov, V. *et al.* (2022) Cell2location maps fine-grained cell types in spatial transcriptomics. *Nat Biotechnol* 40, 661-671. 10.1038/s41587-021-01139-4

14. Allen Reference Atlas - Mouse Brain (2011). Allen Institute for Brain Science

15. Korotkevich, G.E., Sukhov, V., Budin, N., Shpak, B., Artyomov, M. N., Sergushichev, A. (2021) Fast gene set enrichment analysis. *BioRxiv*. doi.org/10.1101/060012

16. Grandjean, J.M.D. *et al.* (2019) Deconvoluting Stress-Responsive Proteostasis Signaling Pathways for Pharmacologic Activation Using Targeted RNA Sequencing. *ACS Chem Biol* 14, 784-795. 10.1021/acschembio.9b00134

17. Rosarda, J.D. *et al.* (2021) Metabolically Activated Proteostasis Regulators Protect against Glutamate Toxicity by Activating NRF2. *ACS Chem Biol* 16, 2852-2863. 10.1021/acschembio.1c00810

18. Aibar, S., Gonzalez-Blas, C. B., Moeman, T., Huynh-Thu, V. A., Imrichova, H., Hulselmans, G., Rambow, F., Marine, J. -C., Geurts, P., Aerts, J., van den Oord, J., Kalender Atak, Z., Wouters, J., Aerts, S. (2017) SCENIC: single-cell regulatory network interference and clustering. *Nature Methods* 14, 1083-1086

19. Hsu, S.M. *et al.* (1981) The use of antiavidin antibody and avidin-biotin-peroxidase complex in immunoperoxidase technics. *Am J Clin Pathol* 75, 816-821. 10.1093/ajcp/75.6.816
